# Hierarchically patterned polyurethane microgrooves featuring nanopillars or nanoholes for neurite elongation and alignment

**DOI:** 10.3762/bjnano.14.96

**Published:** 2023-11-29

**Authors:** Lester Uy Vinzons, Guo-Chung Dong, Shu-Ping Lin

**Affiliations:** 1 Doctoral Program in Tissue Engineering and Regenerative Medicine, National Chung Hsing University, Taichung City 40227, Taiwan (R.O.C.); 2 Institute of Biomedical Engineering and Nanomedicine, National Health Research Institutes, Miaoli County 35053, Taiwan (R.O.C.); 3 Graduate Institute of Biomedical Engineering, National Chung Hsing University, Taichung City 40227, Taiwan (R.O.C.)

**Keywords:** hierarchical, nanopatterning, neurite alignment, neurite outgrowth, topography

## Abstract

Surface micro- and nanostructures profoundly affect the functional performance of nerve regeneration implants by modulating neurite responses. However, few studies have investigated the impact of discrete nanostructures, such as nanopillars and nanoholes, and their combination with microgrooves on neurite outgrowth and alignment. Furthermore, numerous techniques have been developed for surface micro-/nanopatterning, but simple and low-cost approaches are quite limited. In this work, we show that nanopillars and nanoholes, and their combination with microgrooves, can be patterned on polyurethane (PU) films using a low-cost, reusable photoresist master mold prepared via nanosphere lens lithography and UV-LED photolithography, with specific “reinforcement” methods for overcoming the inherent drawbacks of using photoresist masters. We show that the PU nanopillars and nanoholes increase the neurite length of pheochromocytoma 12 (PC12) cells through unique growth cone interactions. Moreover, we demonstrate, for the first time, that hierarchically patterned nano-/microstructured PU films enhance both PC12 neurite elongation and alignment, showing the potential use of our proposed method for the micro-/nanopatterning of polymers for nerve tissue engineering.

## Introduction

The surface features of biomaterials at the micro- and the nanoscale play a crucial role in modulating tissue responses and in determining the functional and temporal efficacy of implants [1]. Micro- and nanoscale surface structures affect cellular functions through micro- and nanometer-sized cell compartments, such as the nucleus, filopodia, and focal adhesions, resulting in the modulation of signal cascades that leads to changes in cell proliferation, attachment, orientation, and differentiation, among others [2]. In nerve tissue engineering, the implant micro- and nanotopography serve as physical cues that promote nerve cell survival, neural stem cell recruitment and differentiation, and axonal guidance and regeneration [3]. The ability of topographical features to guide axons is particularly important in peripheral nerve regeneration, where unidirectional continuous micro-/nanostructures, such as fibers and grooves, in nerve guidance conduits facilitate axonal elongation and guidance and accelerate functional recovery [4].

Aside from continuous structures, different types of discrete micro- and nanostructures in the form of pillars, wires, tubes, holes, and cones have also been shown to positively affect neural functions and neurite outgrowth [3]. Studies on in vitro models for peripheral neurons show promising results for such structures, to wit, poly(3,4-ethylenedioxythiophene) nanotubes and SU-8 nanoholes resulted in significantly longer neurites in pheochromocytoma 12 (PC12) cells [5–6], poly(lactic-*co*-glycolic acid) nanodots enhanced the proliferation and neurite sprouting of Neuro-2a cells [7], and oriented elliptical Si microcones induced alignment and increased fasciculation in rat superior cervical ganglion axons [8]. With their effects complementing those of continuous structures, the question arises: Can discrete structures be combined with continuous structures for possible synergistic effects? Indeed, several studies have fabricated hierarchical discrete nanostructures on continuous microstructures in order to better mimic the micro- and the nanostructure of the native nerve microenvironment. While several of these focused on stem cell differentiation [9–10], a couple of studies explored their effects on axonal guidance. Lee et al. [11] found that nanorough microridges composed of laser-patterned Al/Al_2_O_3_ nanowires increase cell attachment and effectively guide dorsal root ganglia axons. Also, Huang et al. [12] showed that microgrooves with scattered nanodots result in neurite elongation and alignment of spinal cord neurons as well as functional connection between spinal cord slices. Studies on the application of discrete nanostructures and multiscale structures on peripheral nerves are few and far between. Thus, further work is necessary to ascertain the potential of such structures for peripheral nerve regeneration.

One of the reasons for the limited work on discrete nanostructures and hierarchical structures may be the expensive or non-versatile techniques for substrate fabrication. For instance, traditional techniques, such as electron-beam lithography, laser writing, and cleanroom photolithography, have flexibility in design but require costly equipment [13–14]. Relatively cheaper techniques, such as anodization, electroplating, and electrospinning, are limited by the choice of materials and patterns [13]. Other simple techniques, such as nanoimprinting and mold casting are ideal for pattern replication on thermoplastic and soluble polymers; however, they require master molds, which are typically fabricated using the abovementioned traditional techniques [14]. Therefore, there is still a need to develop simple and cost-effective fabrication methods applicable to a wide range of nano- and micropatterns and biomaterials.

In our previous studies, we have shown how nanosphere lens lithography (NLL) can be used with a low-cost ultraviolet light-emitting diode (UV-LED) system to create arrays of nanodots and nanopillars combined with microgrooves on the epoxy-based SU-8 negative photoresist [15–16]. While we found an improvement in PC12 neurite alignment on the ridge areas of nanopillared microgrooves, the overall alignment was not significantly different from that of plain microgrooves and there was a slight decrease in neurite length [16]. In this work, we provide significant advancements to our previous study in three main areas: first, by fabricating a new hierarchical SU-8 structure consisting of nanoholes on microgrooves; second, by demonstrating that the low-cost SU-8 substrates can be used as a reusable master mold to create nano-/micropatterns on polyurethane (PU), a soft, versatile material that has been used for nerve conduits [17]; and finally, by showing, for the first time, significant enhancement of both PC12 neurite elongation and alignment on the hierarchically structured microgrooves featuring nanopillars or nanoholes. Moreover, we found that replica molding using nano-/microstructured photoresist masters is a non-trivial step and requires specific “photoresist reinforcement” strategies to overcome inherent photoresist issues. Overall, our work demonstrates a promising method for the creation of hierarchical nano-/microstructures on various polymers for nerve implant applications.

## Results and Discussion

### Fabrication and characterization of PU nanopillar and nanohole substrates

**Figure 1:**
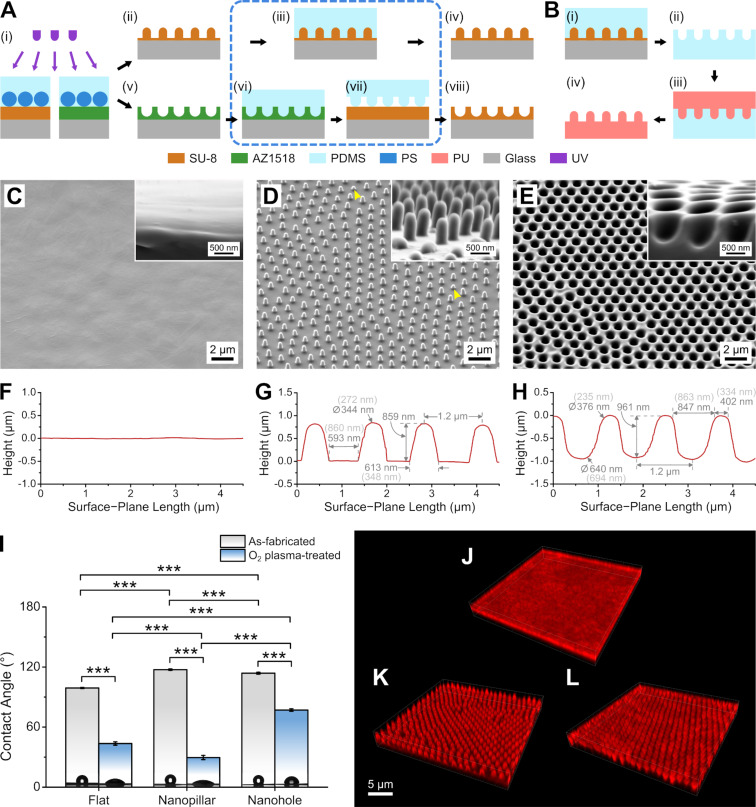
Fabrication process and characteristics of the PU substrates (non-grooved). (A) Preparation of SU-8 nanopillar/hole master molds. (The steps enclosed by the blue dashed box are the “photoresist reinforcement” steps described in the main text.) (B) Replication of nanostructures on PU. (C–E) Scanning electron micrographs (SEM) of PU flat (C), nanopillar (D), and nanohole (E) substrates, with corresponding high-magnification images (insets). (Yellow arrowheads in (D) indicate smaller nanopillars. See further discussion in the text.) (F–H) Cross-sectional profile of the flat (F), nanopillar (G), and nanohole (H) PU surface from atomic force microscopy scans, showing the dimensions of the nanostructures (G, H). (Dimensions in parentheses were obtained from SEM images in Supporting Information File 1, Figure S4.) (I) Water contact angles on the PU films before and after O_2_ plasma treatment (***p < 0.001; *n* = 6). (J–L) 3D confocal fluorescence micrographs of immunostained adsorbed laminin on PU flat (J), nanopillar (K), and nanohole (L) substrates.

We first fabricated nanopillar and nanohole arrays on medical-grade polyether-based PU (Tecothane^®^) to determine whether they have positive effects on PC12 neurite outgrowth. The fabrication process involves first creating the reusable photoresist master molds using NLL with a custom-made UV-LED exposure system [15–16]. This allows for the direct fabrication of a nanopillar array on an SU-8 film (Figure 1A(i) and (ii)). SU-8 generates a strong acid in UV-exposed areas, which, in turn, undergo acid-initiated crosslinking during the post-exposure baking step [18]. However, we found that the subsequent formation of a polydimethylsiloxane (PDMS) inverse mold from the SU-8 nanopillar array fails due to the breakage of the brittle SU-8 nanopillars (Supporting Information File 1, Figure S1A,B), while further hard-baking to strengthen pillar adhesion causes SU-8 reflow, resulting in a dramatic decrease in the pillar aspect ratio (Supporting Information File 1, Figure S1C). Therefore, we employed a unique solution to “reinforce” the SU-8 nanopillars whereby the SU-8 is hard-baked while the nanostructures are encapsulated in cured PDMS (Figure 1A(iii)). This effectively entrapped the reflowing SU-8, allowing for the preservation of the nanopillar structures while further crosslinking the SU-8 (Figure 1A(iv)). This enabled the release of the PDMS film without breakage of the nanopillars (Supporting Information File 1, Figure S1D).

Application of NLL on the positive photoresist AZ1518 allowed for the formation of a nanohole array (Figure 1A(v)). AZ1518 contains a polymerized phenolic resin (Novolak) and a diazonaphthoquinone sulfonate photoactive chemical that is converted into a carboxylic acid upon UV exposure, resulting in increased solubility of the photoresist in the alkaline developer [19]. Nevertheless, the solvent of AZ1518 (and many other photoresists), propylene glycol monomethyl ether acetate, is also the main constituent of the SU-8 developer, rendering AZ1518 incompatible with the subsequent micropatterning step for the hierarchical structures. Therefore, we replicated the AZ1518 nanoholes unto SU-8 using capillary thermal imprinting and then crosslinked the imprinted SU-8 layer (Figure 1A(vi) and (vii)), resulting in a “reinforced” nanohole array (Figure 1A (viii)). Using these “reinforced” photoresist masters, PDMS replica molding and PU solvent casting allowed for the creation of the PU nanopillar and nanohole substrates (Figure 1B and Supporting Information File 1, Figure S2).

Scanning electron microscopy (SEM) images (Figure 1C–E) confirm the featureless surface of flat PU and the ordered arrays of nanopillars and nanoholes on the nanopatterned films. For the PU nanopillar substrate, some short pillars occassionally appeared (Figure 1D), which were also visible under an optical (metallurgical) microscope (Supporting Information File 1, Figure S3A). This was probably due to the polymerization of uncrosslinked PDMS in the mold nanoholes when the PDMS expands during PU casting (Supporting Information File 1, Figure S3B). When lots of short nanopillars had appeared, the PDMS mold was discarded and a new mold was prepared using the SU-8 master. In contrast, we did not observe shallow PU nanoholes on our samples, which was probably due to the more open surface of the PDMS mold nanopillars, resulting in a lesser degree of contact among uncrosslinked PDMS monomers (Supporting Information File 1, Figure S3C).

Atomic force microscopy (AFM) scans of the samples (Figure 1F–H) show that the nanopillars and nanoholes have sub-micrometer feature sizes and a periodicity of around 1.2 µm. Due to AFM measurement artifacts, especially for lateral dimensions of high-aspect-ratio nanostructures [16], we re-measured some dimensions using SEM images (Supporting Information File 1, Figure S4). The nanopillars were around 860 nm high and 350 nm wide at the base and had a rounded tip. The nanoholes were around 960 nm deep and 860 nm wide at the opening and had a rounded bottom. The space between the pillars and holes were around 860 nm and 330 nm, respectively.

The wettability of a surface is a good predictor of protein adsorption and bioactivity [20]. For the extracellular matrix protein laminin, good adsorption and cell growth have been found on hydrophilic, O_2_ plasma-treated substrates [21]. We measured the water contact angle (CA) on the PU samples (Figure 1I) and found that all of the as-fabricated samples were hydrophobic (CA > 90°), with the nanostructured substrates being more so. Since the CA for flat PU indicates a hydrophobic surface, the increase in CA on the nanopillar and nanohole substrates may be due to either a Wenzel- or a Cassie-type of wetting [22]. To improve wetting on the substrates, we treated our samples with mild O_2_ plasma before laminin incubation. After plasma treatment, all samples became hydrophilic (CA < 80°), with the nanopillar substrate having the smallest CAs (CA ≈ 30°). Based on confocal fluorescence microscopy of immunostained samples (Figure 1J–L), laminin successfully adsorbed onto the O_2_ plasma-treated PU samples. There was good laminin coverage on all of the samples, even on the nanostructures, as indicated by the fluorescence patterns conforming to the structure shapes. Laminin was also successfully coated on the flat areas surrounding the pillars and holes, as shown in the corresponding confocal slices in Supporting Information File 1, Figure S5. O_2_ plasma treatment of PU enables strong laminin adsorption possibly due to the introduction of C=O and C–OH bonds on the surface, which leads to a negative charge for electrostatic attraction of positively charged laminin molecules [23–24].

### PC12 neurite outgrowth on PU nanopillars and nanoholes

**Figure 2:**
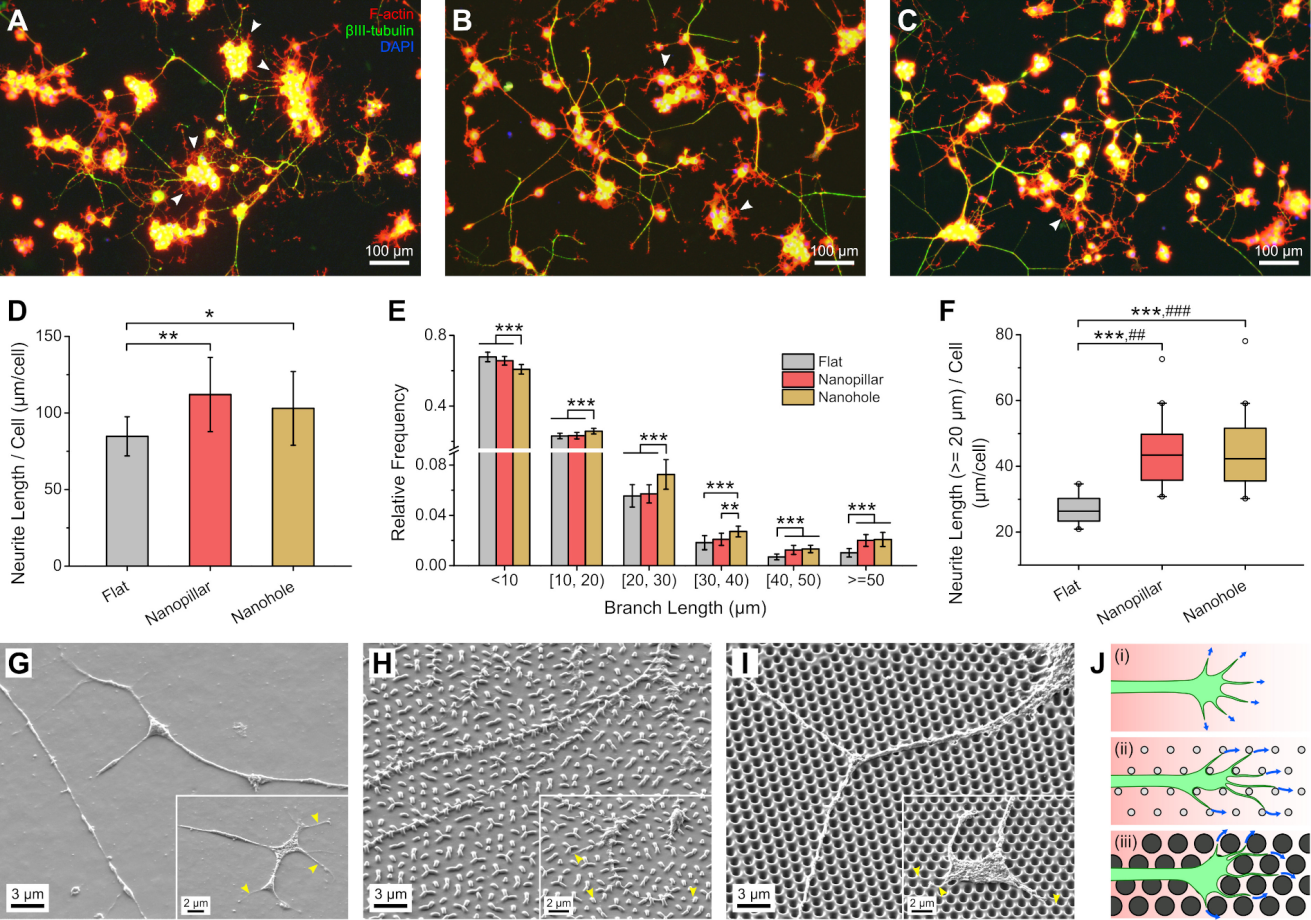
Characteristics of PC12 neurite outgrowth on PU substrates (non-grooved). (A–C) Fluorescence micrographs (merged) of PC12 cells with stained actin, beta-III tubulin, and nucleus on PU flat (A), nanopillar (B), and nanohole (C) substrates. (White arrowheads indicate short projections. Background subtraction and brightness/contrast adjustment were performed. For separate, non-background-subtracted images, please refer to Supporting Information File 1, Figure S7.) (D–F) Quantification of different PC12 neurite parameters: (D) total neurite length per cell, (E) relative frequency histogram of neurite branch length, and (F) total neurite length of branches greater than or equal to 20 µm per cell (*p < 0.05; **,##*p* < 0.01; ***,###*p* < 0.001; *n* = 15). In (F), asterisk (*) and number (#) symbols refer to the distribution and median, respectively. (G–I) Scanning electron micrographs of PC12 neurites and growth cones (insets) on PU flat (G), nanopillar (H), and nanohole (I) substrates. (Yellow arrowheads indicate filopodial projections. In (H), most of the PU nanopillars have collapsed due to drying step prior to SEM.) (J) Hypothesized filopodial behavior during PC12 growth cone movement on the PU flat (i), nanopillar (ii), and nanohole (iii) substrates.

PC12 cells attached well on all the laminin-coated substrates and showed good viability and proliferation on the nanostructured PU films, especially on the PU nanoholes (Supporting Information File 1, Figure S6). After differentiation, PC12 cells extended beta-III tubulin positive neurites, with longer neurites appearing on the nanostructured substrates (Figure 2A–C and Supporting Information File 1, Figure S7) and more short projections emanating from the soma on the flat substrate (white arrowheads). Quantification of neurite length (Figure 2D) confirms increased neurite length per cell on the nanopillar and nanohole substrates, with means at least 1.2× higher than that of flat PU (*p* < 0.05). Analysis of the neurite branch lengths (Figure 2E) also shows that the proportion of branches greater than or equal to 40 µm is bigger on the nanopatterned substrates compared with flat, while the proportion of branches less than 20 µm (i.e., the average soma diameter) is smaller (Supporting Information File 1, Figure S8). Considering only branches greater than or equal to 20 µm, the improvement in neurite length is enhanced, with the median neurite length per cell on the nanopatterned substrates at least 1.6× greater than that of flat PU (*p* < 0.01) (Figure 2F). We did not find any differences in the number of primary neurites per cell among the substrates (Supporting Information File 1, Figure S9A), and the neurite length normalized to the primary neurite count showed similar enhancement on the nanopatterned substrates (Supporting Information File 1, Figure S9B,C). There were also no significant differences in the amount of neurite branching among the nanopatterned substrates and the flat PU (Supporting Information File 1, Figure S10).

The number of cells and primary neurites was lower on the nanopillar substrate compared with flat PU (Supporting Information File 1, Figure S11), probably because of lower proliferation of differentiating PC12 cells on the nanopillars. Correlational analysis shows that the cell and primary neurite counts have a moderate to strong inverse relationship with the neurite length parameters (Supporting Information File 1, Table S1) and thus might have contributed to the increased normalized neurite length on the nanopillar substrate. However, we think that they were not the determining factors as neurites were indeed qualitatively longer on the nanopillar substrate (Figure 2B), and sufficient space was available on the flat substrate for neurite extension despite higher cell and primary neurite counts (Figure 2A).

SEM images of the PC12 cells on PU samples (Figure 2G–I) show that the neurites grew unimpeded on both the flat and nanostructured substrates. On the nanopillar array (Figure 2H), the neurites and neurite tips (presumably growth cones; insets) laid on the flat base area between the pillars and most likely anchored on most of the nanopillar sidewalls, resulting in a more complex, meta-2D or “2.5D” growth environment [25]. (Note that the SEM shows collapsed nanopillars due to the drying process.) Growth cone filopodia also appear to extend towards nanopillars on the flat base areas. On the nanohole array (Figure 2I), the neurites and growth cones passed between and over the holes. However, the growth cone filopodia passed along the spaces and edges between the holes without being suspended across the holes.

The enhancement of PC12 neurite elongation observed here is similar to that previously reported on SU-8 nanoholes (around 250 nm in diameter and 50 nm in spacing) [6] and different from that on gold nanopillars (around 230 nm in diameter and 70 nm in spacing) and nanopores (around 200 nm in diameter and 40 nm in spacing) [26]. Furthermore, in our previous study [16], SU-8 nanopillars with slightly smaller inter-pillar gaps (around 750 nm) than those used here inhibited neurite outgrowth. Thus, it appears that PC12 neurites elongate when continuous narrow pathways of sufficient width are present, as in the nanohole array here and in [6], as well as when the neurites can grow on a flat surface with multiple attachment areas, as in the nanopillar array here (neurites growing on the base area) and as opposed to that in our previous study (neurites growing on top of the pillars) [16] and in [26]. We hypothesize that the nanopillar sidewalls and nanohole spaces act as attachment and guidance cues, respectively, for the growth cone filopodia, facilitating its forward movement and the extension of the neurite, as illustrated in Figure 2J. It will be interesting to determine if such cues provided by the nanopillars and nanoholes could be modulated by the nanopillar/nanohole spacings. This could be the subject of future work utilizing nanosphere lenses with different sizes.

### Fabrication and characterization of PU pillar–groove and hole–groove substrates

**Figure 3:**
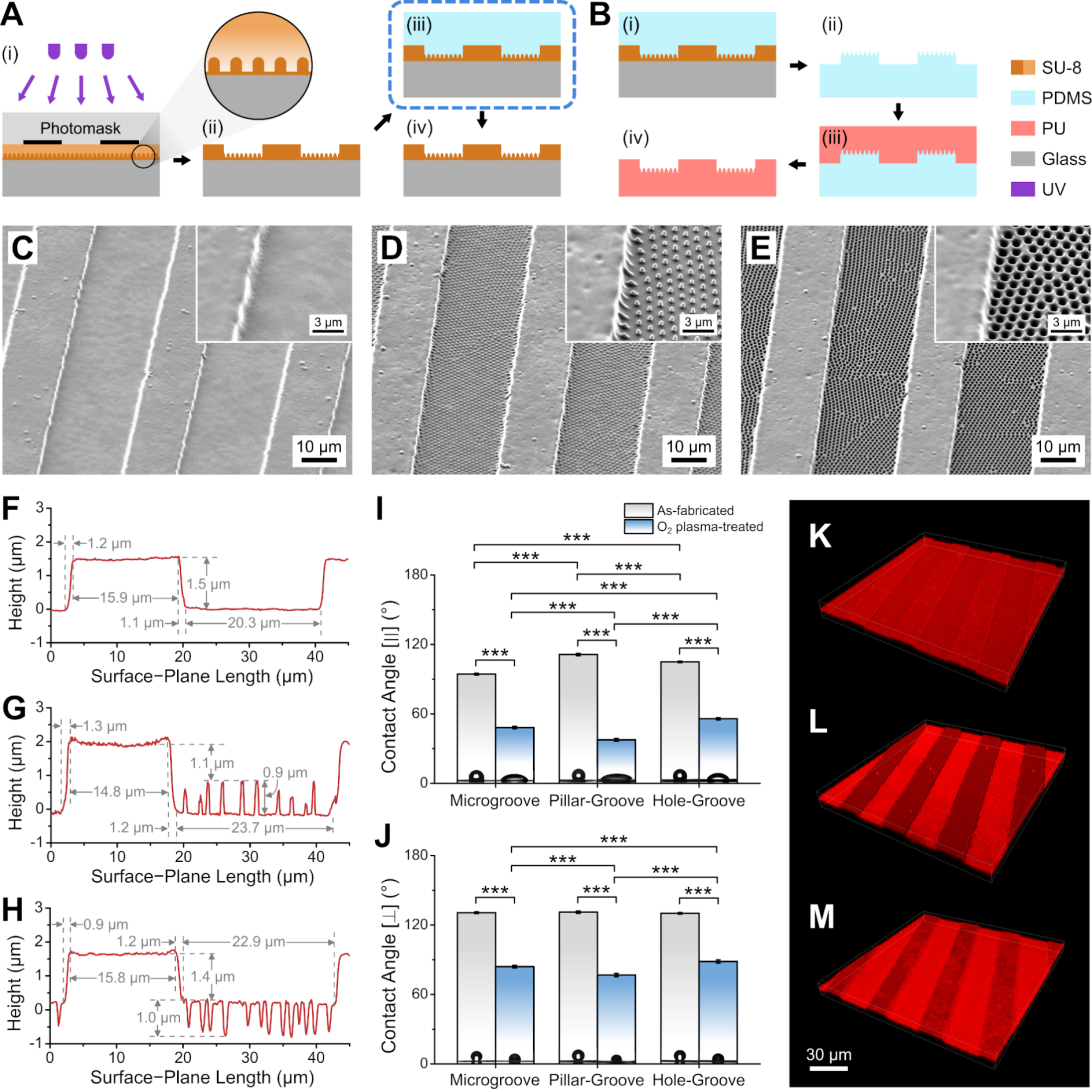
Fabrication process and characteristics of the grooved PU substrates. (A) Preparation of SU-8 nanopillar/hole on microgroove master molds. (The step enclosed by the blue dashed box is the “photoresist reinforcement” step described in the main text.) (B) Replication of nano-/microstructures on PU. (C–E) Scanning electron micrographs of PU microgroove (C), pillar–groove (D), and hole–groove (E) substrates, with corresponding high-magnification images (insets). (F–H) Cross-sectional profile of the microgroove (F), pillar–groove (G), and hole–groove (H) PU surface from atomic force microscopy scans, showing the dimensions of the structures. (I, J) Water contact angles on the PU films before and after O_2_ plasma treatment with the contact angle baseline in parallel with (I) or perpendicular to (J) the groove axis (***p < 0.001; *n* = 6). (K–M) 3D confocal fluorescence micrographs of immunostained adsorbed laminin on PU microgroove (K), pillar–groove (L), and hole–groove (M) substrates.

After confirming that PU nanopillars and nanoholes promote neurite elongation in PC12 cells, we combined the nanostructures with microgrooves to determine their potential use in neurite guidance. Following the strategy in our previous work [16], hierarchical structures in the SU-8 master were achieved using a simple UV-LED photolithography step following NLL (Figure 3A(i) and (ii)). The starting samples for photolithography were a hard-baked SU-8 film, SU-8 nanopillars (not hard-baked), and SU-8 nanoholes for the creation of plain microgrooves (“microgroove”), nanopillars on microgrooves (“pillar–groove”), and nanoholes on microgrooves (“hole–groove”), respectively. (Note that for the nanoholes, the spin-coating of the second SU-8 layer occasionally results in a patchy film, probably because of some residual PDMS from the nanopillar imprinter. Investigation of the SU-8 post-exposure baking parameters during thermal imprinting seems necessary for more consistent results in the future.) As with the SU-8 nanopillar array, a “reinforcement” step consisting of hard-baking in cured PDMS (Figure 3A(iii)) was necessary for the pillar–groove master to prevent breakage of the nanopillars (Figure 3A(iv)).

After PDMS replica molding and PU solvent casting (Figure 3B), the hierarchical patterns were successfully replicated on PU films (Figure 3C–E and Supporting Information File 1, Figure S12). As can be seen from the AFM scans (Figure 3F–H), the microridge areas were around 15 µm in width, while the microgroove areas were slightly wider, around 20–24 µm. The microridge height was 1.4–1.5 µm on the microgroove and hole–groove substrates, while it was 1.1 µm or 2 µm on the pillar–groove substrate if measured to the tip or base of the nanopillars, respectively.

Water CA measurements on the grooved PU samples lead to two distinct CAs on each sample: one with the CA baseline in parallel with the groove axis (CA [∥]; Figure 3I) and another with the CA baseline perpendicular to the groove axis (CA 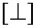; Figure 3J). As can be seen in Figure 3I, the CA [∥] values have similar trends as their non-grooved counterparts (Figure 1I), indicating dominance of the nanostructure properties in this direction. In contrast, the CA 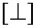 values were higher and remained quite close to each other in magnitude even after O_2_ plasma treatment, signifying the dominance of the microgroove properties in this orientation. Despite the relatively high CA 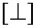 values after plasma treatment, the lower CA [∥] values (<60°) indicate that the surface was hydrophilic enough along the groove direction, and good solution coverage could be achieved during laminin coating.

We also confirmed laminin adsorption on the grooved PU samples using confocal fluorescence microscopy. Figure 3K–M show successful laminin coating of the samples on both the microridge and microgroove areas, with brighter groove areas on the pillar–groove and hole–groove samples (Figure 3L and M) due to the higher effective surface area resulting from the nanostructures.

### PC12 neurite outgrowth on nanopatterned PU microgrooves

**Figure 4:**
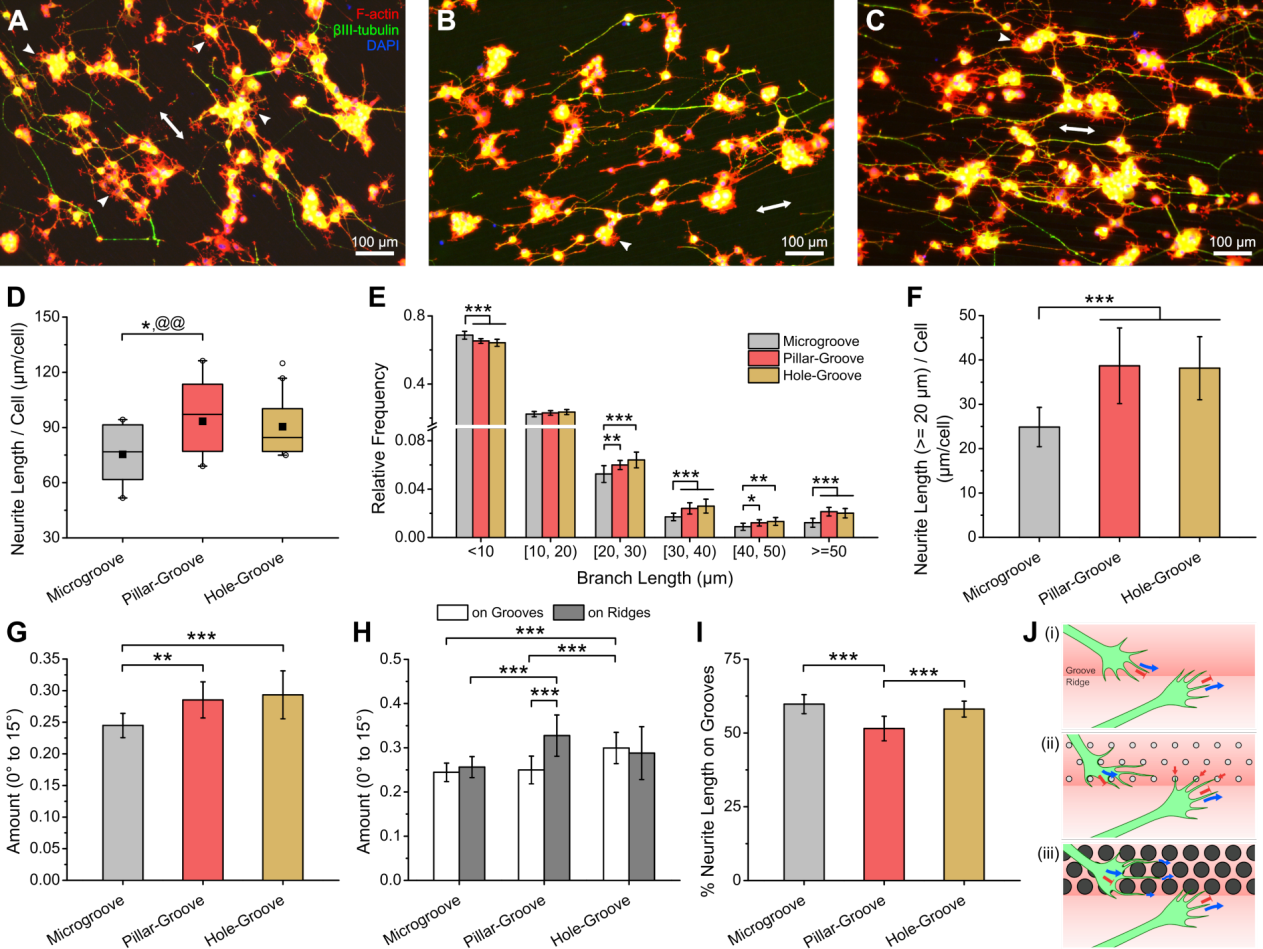
Characteristics of PC12 neurite outgrowth on grooved PU substrates. (A–C) Fluorescence micrographs (merged) of PC12 cells with stained actin, beta-III tubulin, and nucleus on PU microgroove (A), pillar–groove (B), and hole–groove (C) substrates. (White arrowheads indicate short projections, while white double-headed arrows indicate the direction of the grooves. Background subtraction and brightness/contrast adjustment were performed. For separate, non-background-subtracted images, please refer to Supporting Information File 1, Figure S14.) (D–I) Quantification of different PC12 neurite parameters: (D) total neurite length per cell, (E) relative frequency histogram of neurite branch length, (F) total neurite length of branches greater than or equal to 20 µm per cell, (G) relative amount of aligned neurites in entire area, (H) relative amount of aligned neurites on groove and ridge areas, and (I) percentage of neurite length on groove areas (*p < 0.05; **,@@*p* < 0.01; ***p < 0.001; *n* = 15). In (D), the microgroove and pillar–groove substrates have normally distributed data (with the means shown as black-filled squares). The asterisk (*) and at (@) symbols in (D) refer to the distribution and mean, respectively. (J) Hypothesized behavior of PC12 growth cones on the PU microgroove (i), pillar–groove (ii), and hole–groove (iii) substrates.

PC12 cells also attached well on all grooved PU substrates and showed good viability and proliferation on the hierarchically patterned microgrooves, especially on the hole–groove substrate (Supporting Information File 1, Figure S13). After differentiation, PC12 cells extended neurites preferentially in the direction of the microgrooves (Figure 4A–C and Supporting Information File 1, Figure S14). Quantification of neurite extension shows that only the pillar-groove substrate yielded a statistically different distribution of neurite length per cell compared with the microgroove substrate (*p* = 0.049), with a mean around 1.2× higher (*p* = 0.008) (Figure 4D). Nevertheless, there was still a larger proportion of longer neurite branches (≥20 µm) and a smaller proportion of branches less than 20 µm on both the pillar–groove and hole–groove substrates than on the microgroove substrate (Figure 4E and Supporting Information File 1, Figure S15). Considering only branches greater than or equal to 20 μm, the improvement in neurite length was again enhanced, with the mean neurite length per cell on the nanopatterned substrates at least 1.5× greater than that of the microgroove substrate (*p* < 0.001) (Figure 4F). There were no significant differences in the number of primary neurites per cell among the substrates (Supporting Information File 1, Figure S16A), and a similar enhancement of the neurite length on nanopatterned substrates was found when normalized to the primary neurite count (Supporting Information File 1, Figure S16B,C). Moreover, there were also no significant differences in the amount of neurite branching among the substrates (Supporting Information File 1, Figure S17). Similar to the non-grooved substrate, the pillar–groove substrate had lower cell and primary neurite counts (Supporting Information File 1, Figure S18), and moderate to strong negative correlations were found between the cell and primary neurite counts and neurite length parameters (Supporting Information File 1, Table S2), which might have slightly inflated the normalized neurite length values for the pillar–groove substrate.

Quantitative analysis of neurite directionality on the grooved substrates reveals an improvement in neurite alignment on the nanopatterned grooves, as can be seen in the increase in the amount of neurites aligned within 15° of the grooves, considering all neurite branches (Figure 4G and Supporting Information File 1, Figure S19A) and branches greater than or equal to 20 µm only (Supporting Information File 1, Figure S19B). In particular, the amount of aligned neurites was at least 1.17× higher on the nanopatterned substrates compared to the plain microgrooves (*p* < 0.01). Further analysis of the neurite directionality on the ridge and groove areas (Supporting Information File 1, Figure S20) reveals that the enhancement of neurite alignment on the pillar–groove and hole–groove substrates was due to the improvement in alignment on the ridge areas for the former and on groove areas for the latter (Figure 4H). Moreover, the improvement in neurite alignment on the ridges of the pillar–groove substrate was accompanied by an overall decrease in neurite localization on the grooves (i.e., an increase in neurites on ridges); however, no increase in neurite localization on the grooves in the hole–groove substrate was observed (Figure 4I). This change in neurite localization seems not to be mainly caused by the location of the soma as only an increase in soma localization on the groove areas for the hole–groove substrate was found (Supporting Information File 1, Figure S21).

The alignment of neurites on microgrooves has been mainly attributed to the bending rigidity of the neurite cytoskeleton, leading to the resistance of the growth cone to cross groove steps (Figure 4J(i)) [27–28]. Here, we observe that the microgrooves with a width of around 20 µm and a depth of around 1.4–2 µm are sufficient to elicit good alignment of PC12 neurites, consistent with previous studies [29–30]. Meanwhile, the improved neurite alignment on the pillar–groove substrate was probably due to the failure of the growth cone filopodia to establish stable adhesions on the highly discontinuous pillar tips [16], resulting in the retraction of the growth cone (Figure 4J(ii)) and the increased confinement of the neurites on the ridge areas. Although the ridges were slightly higher on the pillar–groove substrate (when measured from the pillar base), this was probably not the main determining factor since no improved alignment on the groove areas was observed. The enhanced neurite alignment on the hole–groove substrate could be due to the guidance effect of the submicrometer-wide spaces between the holes on the growth cone filopodia, amplifying the guidance effect of the steps on the groove areas (Figure 4J(iii)).

## Conclusion

We have developed a low-cost method for the creation of nanopillar or nanohole arrays and hierarchical structures consisting of nanopillar/holes on microgrooves on PU films for the enhancement of neurite elongation and alignment. The fabrication process involves the use of NLL and UV-LED photolithography for master mold preparation and soft lithography and solvent casting for PU film patterning. Challenges in the use of photoresist master molds for PDMS replica molding and microgroove formation were addressed using “reinforcement” strategies. Differentiation of PC12 cells on the PU substrates resulted in longer neurites on the nanopillar and nanohole arrays. Furthermore, when combined with microgrooves, the discrete nanostructures enhanced not only neurite elongation but also neurite alignment as compared with a plain microgrooved PU substrate. The low-cost method presented in this study facilitates the creation of nano-/microstructures on substrates of different solvent-castable polymers without the use of expensive equipment. Moreover, the hierarchically patterned microgrooves featuring nanopillars and nanoholes provide an additional strategy for the enhancement of next-generation nerve guidance conduits.

## Experimental

### Materials

Polystyrene nanospheres (ca. 1.1 µm diameter), laminin from Engelbreth–Holm–Swarm murine sarcoma, and nerve growth factor (2.5S, from murine submaxillary glands) were purchased from Sigma, Merck KGaA (Germany). SU-8 50 and SU-8 developer were obtained from Kayaku Advanced Materials (MA, USA), while AZ1518 and AZ 300 MIF developer were purchased from MicroChemicals GmbH (Germany). PDMS, γ-butyrolactone (GBL), and PU pellets (Tecothane^®^, clear; TT-1085A) were obtained from Sil-More Industrial Ltd. (Taiwan), Echo Chemical Co., Ltd. (Taiwan), and Lubrizol Advanced Materials, Inc. (OH, USA), respectively. Dimethylacetamide (Alfa Aesar), phosphate-buffered saline (PBS), sera, rhodamine–phalloidin (RP), 4′,6-diamidino-2-phenylindole (DAPI), and Alexa Fluor 488–beta-III tubulin antibody (AF488-anti-β3 tubulin) were obtained from Thermo Fisher Scientific (MA, USA). RPMI 1640 medium, sodium pyruvate, and HEPES buffer were purchased from Corning (NY, USA). The reusable polystyrene nanosphere lens array (1.1 µm) embedded in PDMS for UV-light focusing was prepared according to procedures described in our previous study [16].

### Preparation of nano-/micropatterned SU-8 master molds

The formation of the SU-8 nanopillar array was similar to that of our previous study [16]. Briefly, a thin SU-8 layer was first hard-baked on a 2.5 cm × 2.5 cm glass slide as an adhesion layer. Then, the SU-8 layer to be patterned was spin-coated using a GBL-diluted SU-8 solution (SU-8 50/GBL vol. ratio 1:0.7) at 5000 rpm and soft-baked at 95 °C for 160 s. An array of polystyrene nanospheres (1.1 µm) embedded in PDMS was placed in conformal contact with the SU-8, and then exposure was performed at a dose of 35–42 mJ·cm^−2^ (Figure 1A(i)). (Older PS-NS/PDMS films seem to require slightly higher UV doses, maybe because of UV oxidation of the films or changes in the shape of the PS-NS caused by residual GBL in the SU-8.) The SU-8 was subjected to a post-exposure bake at 95 °C for 2 min, followed by development in the SU-8 developer for 1 min, rinsing with isopropyl alcohol, and N_2_ drying (Figure 1A(ii)). To prevent breakage of the SU-8 nanopillars during PDMS molding, a SU-8 “reinforcement” step was performed, which entailed hard-baking the photoresist with an encapsulating cured PDMS (A/B wt. ratio 15:1) (Figure 1A(iii)). The hard-baking process was as follows: 65 °C for 5 min, 95 °C for 5 min, 150 °C for 15 min, 170 °C for 1 h, and 195 °C for 1 h. Afterwards, the sample was allowed to cool down to room temperature (RT), and then the PDMS was peeled off (Figure 1A(iv)).

To form the “reinforced” SU-8 nanohole mold, a nanohole template was first formed on the positive photoresist AZ1518, which served as a template for creating a PDMS nanopillar structure for the capillary thermal imprinting of SU-8. An AZ1518 film was spin-coated on glass coverslips at 5000 rpm and soft-baked at 100 °C for 1.5 min. Exposure was performed at a dose of 13 mJ·cm^−2^ with an array of 1.1 μm polystyrene nanospheres (in PDMS) in conformal contact with the photoresist (Figure 1A(i)). AZ1518 was developed with AZ 300 MIF developer for 30 s, followed by rinsing with ultrapure water and N_2_ drying (Figure 1A(v)). The nanopatterned AZ1518 was finally post-baked at 120 °C for 2 min to improve substrate adhesion. PDMS (A/B wt. ratio 10:1) was poured over the nanopatterned AZ1518, degassed, cured at 65 °C on a hotplate overnight, and peeled off to obtain PDMS nanopillars (Figure 1A(vi)).

For imprinting nanoholes on SU-8, a diluted SU-8 solution (SU-8 50/GBL vol. ratio 1:0.37) was spin-coated on a 2.5 cm × 2.5 cm glass slide at 5000 rpm and soft-baked at 95 °C for 2 min. The PDMS nanopillar array was placed in conformal contact with the SU-8, and then the sample was baked at 95 °C for 5 min for thermal reflow of the SU-8 (Figure 1A(vii)). The sample was allowed to cool down to RT and then was flood-exposed at a dose of 180 mJ·cm^−2^. Afterwards, the sample was subjected to post-exposure bake and hard bake via a stepwise increase in temperature: 95 °C for 3 min, 150 °C for 15 min, and 165 °C for 1 h. After baking, the sample was allowed to cool down to RT, and then the PDMS nanopillar film was peeled off (Figure 1A(viii)).

The fabrication process for the grooved SU-8 molds was similar to that of our previous study (Figure 3A(i) and (ii)) [16]. Briefly, the starting samples were 2.5 cm × 2.5 cm glass slides with a hard-baked SU-8 film, SU-8 nanopillars (not hard-baked), and SU-8 nanoholes for the creation of SU-8 microgroove, pillar–groove, and hole–groove substrates, respectively. For the SU-8 microgroove and pillar–groove samples, a SU-8 50/GBL volume ratio of 1:0.37 was used with the following processing parameters: spin speed of 5000 rpm, soft bake at 95 °C for 3 min, UV dose of 200 mJ·cm^−2^, post-exposure bake at 95 °C for 2.5 min, and development for 60 s and 70 s for microgroove and pillar–groove, respectively. A less dilute SU-8 solution with SU-8 50/GBL volume ratio of 1:0.33 was used for the hole–groove sample to create microgrooves of similar depth with the following adjusted parameters: soft bake at 95 °C for 190 s, UV dose of 210 mJ·cm^−2^, post-exposure bake at 95 °C for 160 s, and development for 80 s. The SU-8 pillar–groove sample was also subjected to a hard-baking “reinforcement” step similar to that of the SU-8 nanopillars, as described above (Figure 3A(iii) and (iv)).

All photoresist exposure steps were performed using a custom-made UV-LED system [15], while all baking steps were done on a hotplate.

### Preparation of nano-/micropatterned PU films

PDMS inverse molds were prepared using the SU-8 master molds by pouring PDMS (A/B wt. ratio 10:1) onto the molds, degassing, and curing at 65 °C on a hotplate overnight (Figure 1B(i) and (ii), Figure 3B(i) and (ii)). A flat PDMS mold was also prepared using the top side of a cured PDMS sheet. PDMS rings were placed on the molds to create a well to hold the PU solution during casting.

The PU samples were prepared by solvent casting onto the PDMS molds (Figure 1B(iii) and 3B(iii)). To facilitate filling of the nano- and microstructures, a dilute 5 wt % PU solution in dimethylacetamide was first cast into the molds twice, with slow, partial drying at 80 °C in an oven for 10 min and 15 min in the first and second casting, respectively. Then, to increase the sample thickness for easier handling, a less dilute 10 wt % PU was cast over the partially dried PU, slowly dried in the oven at 80 °C for 10 min, and then fully dried in the oven at 100 °C for 3 h. Afterwards, the samples were allowed to cool down to RT, and then the PU films were carefully peeled off from the molds and cut to form the final samples (Figure 1B(iv) and 3B(iv)).

The PU substrates were characterized using SEM, AFM, and water CA measurements, as described in detail in Supporting Information File 1.

### PC12 culture and neurite outgrowth experiment

PC12 cells (ATCC CRL-1721) were cultured in RPMI 1640 medium (with ʟ-glutamine and sodium bicarbonate), supplemented with HEPES buffer (25 mM), sodium pyruvate (1 mM), heat-inactivated horse serum (10% v/v), fetal bovine serum (5% v/v), and penicillin/streptomycin (100 U·mL^−1^/100 µg·mL^−1^) in a humidified CO_2_ incubator (37 °C, 5% CO_2_).

The PC12 neurite outgrowth experiment was divided into two parts: The first part was neurite outgrowth on PU flat, nanopillar, and nanohole substrates; the second part was neurite outgrowth on PU microgroove, pillar–groove, and hole–groove substrates. The samples were treated with O_2_ plasma (30 W, 20 sccm O_2_, 30 s) to make the PU surface hydrophilic and enhance the adsorption of laminin. The samples were then placed in a 24-well culture plate, sterilized with UV in a biosafety cabinet for 1 h, and coated with 10 µg·mL^−1^ laminin in Ca^2+^/Mg^2+^-free PBS (1 mL per well) at 4 °C overnight. After coating, the substrates were washed twice with Ca^2+^/Mg^2+^-free PBS.

PC12 cells were seeded on the PU samples at 13 × 10^3^ cells per well. After overnight incubation in growth medium, the medium in each well was replaced with 1 mL differentiation medium, which was composed of RPMI 1640 medium (with ʟ-glutamine and sodium bicarbonate), supplemented with HEPES buffer (25 mM), sodium pyruvate (1 mM), heat-inactivated horse serum (1% v/v), penicillin/streptomycin (100 U·mL^−1^/100 µg·mL^−1^), and nerve growth factor (100 ng·mL^−1^). The cells were differentiated for six days, with half of the medium being replaced with fresh differentiation medium every two days. The neurite outgrowth experiments were performed in triplicate.

The adsorbed laminin on the PU substrates was observed using confocal fluorescence microscopy. PC12 neurite outgrowth after differentiation was characterized using fluorescence micrographs of cells stained with RP, AF488-anti-β3 tubulin, and DAPI. Quantification of neurite parameters were performed using a semi-automatic method described in our previous study [16]. SEM was also performed to observe the neurite morphology. Further details on the characterization procedures and statistical analyses can be found in Supporting Information File 1.

## Supporting Information

File 1 Additional details of experimental methods and supplementary data.

## References

[1] Harawaza K, Cousins B, Roach P, Fernandez A (2021). Mater Today Bio.

[2] Leclech C, Villard C (2020). Front Bioeng Biotechnol.

[3] Yang C-Y, Huang W-Y, Chen L-H, Liang N-W, Wang H-C, Lu J, Wang X, Wang T-W (2021). J Mater Chem B.

[4] Ma Y, Gao H, Wang H, Cao X (2021). J Mater Chem B.

[5] Chen H-l, Tian G-z, Yan H, Yang S-x, Kim D-H (2022). Electrochim Acta.

[6] Kim E, Yoo S-J, Kim E, Kwon T-H, Zhang L, Moon C, Choi H (2016). Nanotechnology.

[7] Mimiroglu D, Yanik T, Ercan B (2022). J Biomed Mater Res, Part A.

[8] Simitzi C, Efstathopoulos P, Kourgiantaki A, Ranella A, Charalampopoulos I, Fotakis C, Athanassakis I, Stratakis E, Gravanis A (2015). Biomaterials.

[9] Yang K, Jung H, Lee H-R, Lee J S, Kim S R, Song K Y, Cheong E, Bang J, Im S G, Cho S-W (2014). ACS Nano.

[10] Poudineh M, Wang Z, Labib M, Ahmadi M, Zhang L, Das J, Ahmed S, Angers S, Kelley S O (2018). Nano Lett.

[11] Lee J, Schwarz L K, Akkan C K, Miró M M, Abad O T, Schäfer K-H, Veith M, Aktas C (2013). Phys Status Solidi A.

[12] Huang Y, Jiang Y, Wu Q, Wu X, An X, Chubykin A A, Cheng J-X, Xu X-M, Yang C (2018). ACS Biomater Sci Eng.

[13] Farzam M, Beitollahpoor M, Solomon S E, Ashbaugh H S, Pesika N S (2022). Biomimetics.

[14] Zhang K, Xiao X, Wang X, Fan Y, Li X (2019). J Mater Chem B.

[15] Vinzons L U, Lin S-P (2021). Nanotechnology.

[16] Vinzons L U, Lin S-P (2022). ACS Appl Nano Mater.

[17] Lee T-H, Yen C-T, Hsu S-h (2020). ACS Biomater Sci Eng.

[18] Kayaku Advanced Materials (2020). SU-8 Permanent Negative Epoxy Photoresist Formulations 50–100.

[19] MicroChemicals GmbH Composition and Properties of AZ® and TI Photoresists.

[20] Cai S, Wu C, Yang W, Liang W, Yu H, Liu L (2020). Nanotechnol Rev.

[21] Huang Y-C, Huang C-C, Huang Y-Y, Chen K-S (2007). J Biomed Mater Res, Part A.

[22] Duta L, Popescu A C, Zgura I, Preda N, Mihailescu I N, Mahmood A (2015). Wettability of Nanostructured Surfaces. Wetting and Wettability.

[23] Lin J-H, Chang H-Y, Kao W-L, Lin K-Y, Liao H-Y, You Y-W, Kuo Y-T, Kuo D-Y, Chu K-J, Chu Y-H (2014). Langmuir.

[24] Wilson D J, Rhodes N P, Williams R L (2003). Biomaterials.

[25] Prestwich G D (2007). J Cell Biochem.

[26] Haq F, Anandan V, Keith C, Zhang G (2007). Int J Nanomed.

[27] Li N, Folch A (2005). Exp Cell Res.

[28] Chua J S, Chng C-P, Moe A A K, Tann J Y, Goh E L K, Chiam K-H, Yim E K F (2014). Biomaterials.

[29] Yao L, Wang S, Cui W, Sherlock R, O’Connell C, Damodaran G, Gorman A, Windebank A, Pandit A (2009). Acta Biomater.

[30] Cai L, Zhang L, Dong J, Wang S (2012). Langmuir.

