# Supplementary material for: Hierarchically patterned polyurethane microgrooves featuring nanopillars or nanoholes for neurite elongation and alignment
Source: Beilstein J Nanotechnol. 2023 Nov 29;14:1157–68. doi: 10.3762/bjnano.14.96 (PMC10701984; doi:10.3762/bjnano.14.96)
Supplement: File 1 — Additional details of experimental methods and supplementary data. [file Beilstein_J_Nanotechnol-14-1157-s001.pdf]

BEILSTEIN JOURNAL OF NANOTECHNOLOGY

Supporting Information

for

Hierarchically patterned polyurethane microgrooves featuring nanopillars or nanoholes for neurite elongation and alignment

Lester Uy Vinzons, Guo-Chung Dong and Shu-Ping Lin

Beilstein J. Nanotechnol. **2023,** *14,* 1157–1168. doi:10.3762/bjnano.14.96

Additional details of experimental methods and supplementary data

License and Terms: This is a supporting information file under the terms of the Creative Commons Attribution License (https://creativecommons.org/licenses/by/4.0). Please note that the reuse, redistribution and reproduction in particular requires that the author(s) and source are credited and that individual graphics may be subject to special legal provisions.
The license is subject to the *Beilstein Journal of Nanotechnology* terms and conditions: (https://www.beilstein-journals.org/bjnano/terms)

Characterization of polyurethane (PU) surface morphology

The surface morphology of the nano-/micropatterned PU films was observed using field-emission scanning electron microscopy (FE-SEM; ZEISS Ultra Plus; ZEISS, Germany) and atomic force microscopy (AFM; DI-3100; Veeco, NY, USA). For FE-SEM, the samples were sputter-coated with Au or Pt and then observed in SE2 mode with an accelerating voltage of 3 kV. For AFM, scanning was performed in soft tapping mode with a silicon, tetrahedral probe tip with a radius of curvature < 7 nm, a height of 12–16 μm, and angles of 0° front / 35° back / < 9° side (OPUS 3XC-NA; MikroMasch, Innovative Solutions Bulgaria Ltd.). The scan parameters were as follows: 5 μm × 5 μm or 50 μm × 50 μm scan size, 0.2–0.6 Hz scan rate, 256 lines, and 512 samples/line. AFM data were analyzed using NanoScope Analysis software (version 1.70; Bruker Corp., MA, USA).

Characterization of PU surface wettability

Water contact angle (CA) measurements on the different PU films were performed using a CA goniometer (OCA20 CA system with SCA20 software version 4.3.19; DataPhysics Instruments GmbH, Germany) in static mode with a 2-μL drop of ultrapure water. Determination of the CA's from the water drop images was accomplished using tangent–leaning fitting algorithm in the software. CA's were obtained from two samples of each substrate type, at three random areas of each sample, before and after O_2 plasma treatment.

Characterization of distribution of adsorbed laminin on the PU films

The distribution of adsorbed laminin on the PU substrates was characterized using a confocal laser-scanning microscopy system (FLUOVIEW FV3000; version 2.6.1.243; Olympus Corp., Japan) and immunostaining. The PU substrates were treated with O_2 plasma, sterilized with UV, coated with laminin, and rinsed, following the same procedures in the PC12 culture and

neurite outgrowth experiment. Before immunostaining, the adsorbed laminin was lightly fixed with neutral-buffered formalin (NBF; Sigma, Merck KGaA, Germany) diluted to 5% by phosphate-buffered saline (PBS) for 5 min at room temperature (RT). After rinsing with PBS, the samples were blocked with 1% w/v bovine serum albumin (BSA) in PBS with 0.1% Tween 20 (PBSt) for 30 min at RT. The samples were then incubated with the rabbit laminin antibody solution (2 μg mL^{-1} in 1% BSA-PBSt; Thermo Fisher Scientific, MA, USA) for 2 h at RT. The samples were rinsed with PBS and then incubated with tetramethylrhodamine-conjugated anti-rabbit secondary antibody (4 μg mL^{-1} in 1% BSA-PBSt; Thermo Fisher Scientific, MA, USA) for 2 h at RT. After rinsing with PBS, the samples were stored in PBS at 4°C until ready for confocal imaging.

Confocal fluorescence microscopy was performed using 100× (oil-immersion; 1.4 NA) and 60× (water-immersion; 1.2 NA) objective lenses with software zoom of ×5 and ×1.5, respectively, resulting in corresponding scan sizes of 25.456 μm × 25.456 μm and 141.421 μm × 141.421 μm. In order to visualize the laminin distribution in 3D, confocal *z*-stack images with thicknesses of 3.3–3.5 μm (0.1 μm per slice; 100× objective) and 11.5–14.5 μm (0.5 μm per slice; 60× objective) were obtained, and then 3D images were reconstructed using Imaris software (Bitplane; version 9.3.1; Oxford Instruments, UK).

PC12 proliferation on the PU films

PU samples were prepared and seeded with PC12 cells as described in the PC12 culture and neurite outgrowth experiment. Briefly, the samples were treated with O_2 plasma, coated with laminin, and seeded with 13 × 10^3 cells per well. However, instead of changing to differentiation medium after overnight incubation, the cells were continuously grown in complete growth medium. After one, three, and five days of culture, the cells were washed with PBS once, fresh growth medium was added, and then brightfield images of live cells in random

areas of the samples were taken using an inverted microscope (Leica DM IL LED Fluo; Leica Microsystems, Germany) with a 10× objective. For the determination of the cell counts, the cell areas were manually traced and measured using the ROI Manager tool of ImageJ (version 1.52a; National Institutes of Health, USA). Then, the total cell number per image was determined by dividing the sum of the cell areas by the average area of a single cell measured from five cells in the same image. Three replicates of the experiment were performed. For each replicate, three images for each sample for each measurement day were utilized for the analysis.

Fluorescence microscopy of differentiated PC12 cells

The differentiated PC12 cells on the PU films were visualized by fluorescence staining of F-actin and beta-III tubulin using rhodamine–phalloidin (RP) and Alexa Fluor 488–beta-III tubulin antibody (AF488-anti-β3 tubulin), respectively, with nuclei counterstaining using 4',6-diamidino-2-phenylindole (DAPI). PC12 cells were fixed after six days of differentiation using 10% NBF at RT, rinsed with PBS, and stored in PBS at 4°C until ready for staining. Prior to staining, the cells were permeabilized with 0.25% v/v Triton X-100 in PBS for 10 min, followed by PBS rinsing and blocking with 1% w/v BSA-PBSt. The cells were then incubated with AF488-anti-β3 tubulin (1 μg mL^{-1} in 1% BSA-PBSt) at 4°C overnight. The following day, the cells were stained with RP (0.4 U mL^{-1}) and DAPI (1 μg mL^{-1}) in 1% BSA-PBSt for 1 h at RT, followed by 1% BSA-PBSt wash and a final PBS wash. The stained cells were stored in PBS at 4°C until ready for fluorescence observation.

Fluorescence microscopy was performed using an inverted fluorescence microscope (Leica DMI3000 B; Leica Microsystems, Germany) equipped with a microscope digital camera and imaging software (DP74 and cellSens Standard version 1.17, respectively; Olympus Corp., Japan). For each PU sample, RP, AF488-anti-β3 tubulin, and DAPI images of PC12 cells in five random areas were captured using a 10× objective.

Quantification of PC12 neurite outgrowth

The quantification of the PC12 neurite outgrowth was performed for each captured area (a set of RP, tubulin, and DAPI images) in a semi-automatic manner using plugins in ImageJ and Fiji/ImageJ [1] (version 2.9.0/1.53t), as described in our previous study [2], with some modifications. First, RP, tubulin, and DAPI images were enhanced using rolling-ball background subtraction and brightness/contrast adjustment in ImageJ. (In order to improve neurite tracing, the intensely bright somas in RP images were removed by setting the rolling ball radius sufficiently low.) Merged RP and tubulin images were used to obtain neurite traces (skeletonized image of the neurites) using the NeuriteTracer plugin [3], while merged tubulin and DAPI images were used to detect the soma areas using NeurphologyJ plugin [4], after manual setting of image thresholds. A corrected neurite tracing was obtained by removing the extraneous neurite traces in the soma areas using the NeurphologyJ soma file. For tracings from grooved samples, tracing artifacts due to some fluorescent groove edges were manually removed from the corrected tracing image by erasing the erroneous traces in an image-editing software (GIMP version 2.10.6; GNU General Public License). The total neurite length was determined from the corrected neurite tracing. Furthermore, using the AnalyzeSkeleton plugin and the corrected tracing file, we obtained the branch and junction counts and branch length information for the determination of the branch length distribution. The branch length information also allowed the determination of the total neurite length for branches ≥ 20 μm. To obtain the primary neurite count, the number of neurite attachment points to the soma was determined using NeurphologyJ and the NeuriteTracer tracing output. As with the neurite traces, attachment point artifacts in grooved samples arising from some fluorescent groove edges were manually removed using GIMP software. Cell count was determined by manually counting the nuclei in the DAPI image with the aid of the Multipoint tool of ImageJ.

In order to characterize the alignment of the neurites on the grooved substrates, we dilated the corrected tracings and analyzed them using Fourier components method of Fiji's Directionality plugin (360 bins; 0° to 179.5°). (Dilation was necessary to minimize the directional bias, as discussed in our previous study [2].) The angles were then adjusted to the range of −90° to 89.5°, setting 0° as the direction of the grooves. In order to characterize the alignment of neurites ≥ 20 μm only, we broke up the neurite traces into separate branches by removing the junction pixels using the tagged skeleton image from AnalyzeSkeleton and then used the Analyze Particles tool of ImageJ to retain only the branches ≥ 20 μm; the Directionality plugin was then utilized as before.

As in our previous study [2], neurite alignment, neurite length, and soma area on groove and ridge areas were also separately characterized. The approach was similar where a ridge mask composed of black stripes was created in a vector graphics software (Inkscape version 0.92.4; GNU General Public License) using the corresponding brightfield image of the substrate area as a guide. (The groove lines are visible in the brightfield image and the ridge areas are identifiable as they are slightly narrower than the grooves.) Inversion of the ridge mask results in the groove mask. A binary image containing neurite traces in grooves (or ridges) was obtained by performing an AND operation of the neurite tracing file with the ridge (or groove) mask. The same applies for the soma area file. After obtaining the masked images, neurite length and alignment were determined as described above, while soma area was measured using the Measure tool of ImageJ.

SEM observation of PC12 cells on PU films

SEM observation of formaldehyde-fixed PC12 cells on PU substrates was carried out by first dehydrating the cells in a graded series of ethanol solutions (25%, 50%, 75%, 95%, and 99.9% ×3; 20 min in each) and then sputter-coating the samples with a thin Pt layer. The cells were

then observed using an FE-SEM (ZEISS Ultra Plus) in SE2 mode with an accelerating voltage of 3 kV.

Statistical analysis

Statistical analyses of data were performed using SPSS Statistics software (version 20; IBM Corp., NY, USA). Normality of data and homogeneity of variance were determined by Shapiro–Wilk test and Levene's test, respectively. If the data was normally distributed, one-way analysis of variance (ANOVA) was used to compare groups with Tukey HSD or Games–Howell post-hoc test for data with homegeneous or heterogeneous variances, respectively. When only two groups were being compared, independent samples t-test was used. If the data was not normally distributed, Kruskal–Wallis and median nonparametric tests were utilized. The null hypothesis was rejected if the p value was less than 0.05. Normally distributed data are presented as column plots of the mean ± standard deviation, while non-normally distributed data are presented as box plots showing the median and interquartile range, unless otherwise indicated.

Table S1: Spearman's rank-order correlational analysis of some PC12 neurite parameters on non-grooved PU substrates (n = 45).

Parameters		Spearman's correlation coefficient, ρ	p value
Cell count	Neurite length / cell	−0.676	0.000
Cell count	Neurite length (≥20 µm) / cell	−0.641	0.000
Cell count	Neurite length / primary neurite	−0.442	0.002
Cell count	Neurite length (≥20 µm) / primary neurite	−0.504	0.000
Primary neurite count	Neurite length / cell	−0.331	0.026
Primary neurite count	Neurite length (≥20 µm) / cell	−0.429	0.003
Primary neurite count	Neurite length / primary neurite	−0.525	0.000
Primary neurite count	Neurite length (≥20 µm) / primary neurite	−0.559	0.000

Table S2: Spearman's rank-order correlational analysis of some PC12 parameters on grooved PU substrates (n = 45).

Parameters		Spearman's correlation coefficient, ρ	p value
Cell count	Neurite length / cell	−0.534	0.000
Cell count	Neurite length (≥20 µm) / cell	−0.583	0.000
Cell count	Neurite length / primary neurite	−0.475	0.001
Cell count	Neurite length (≥20 µm) / primary neurite	−0.521	0.000
Primary neurite count	Neurite length / cell	−0.088	0.566
Primary neurite count	Neurite length (≥20 µm) / cell	−0.039	0.799
Primary neurite count	Neurite length / primary neurite	−0.523	0.000
Primary neurite count	Neurite length (≥20 µm) / primary neurite	−0.471	0.001

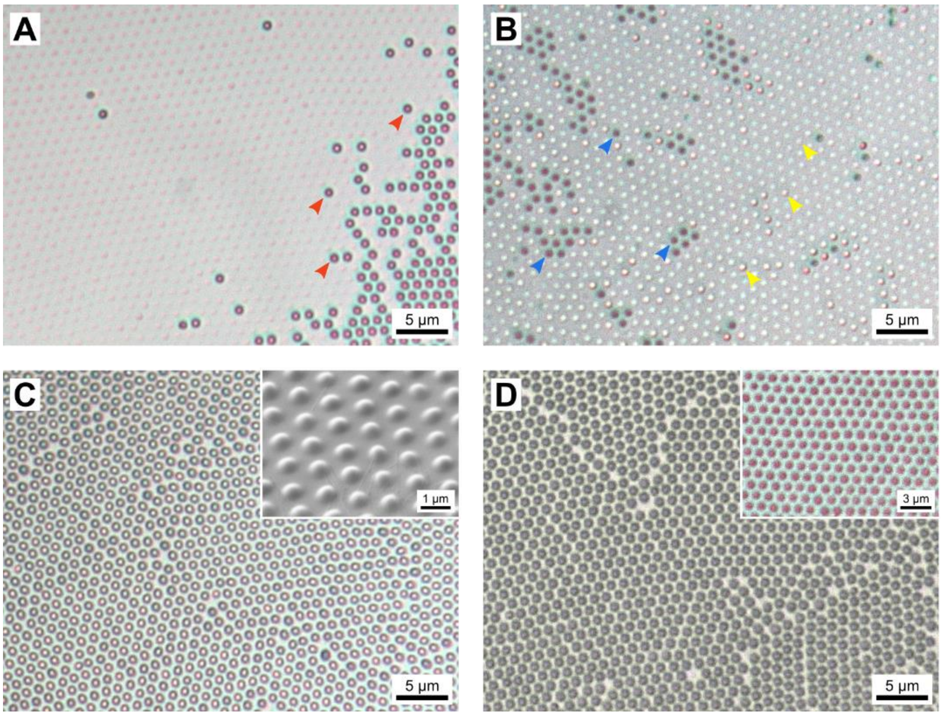

Figure S1: Micrographs depicting PDMS replica molding issues on SU-8 nanopillar array: (A) SU-8 surface showing mostly detached nanopillars after PDMS peel-off and few remaining nanopillars (red arrowheads); (B) PDMS film peeled off from SU-8 nanopillar array showing mostly embedded SU-8 nanopillars (yellow arrowheads) and some hollow nanoholes (blue arrowheads); (C) less prominent structures on SU-8 surface after hard-baking of the nanopillar array (inset shows SEM image of replicated PU structure); and (D) intact SU-8 nanopillars after hard-baking with encapsulating cured PDMS film (inset shows hollow PDMS nanoholes after peel-off). Optical micrographs were taken using BA310MET metallurgical microscope (Motic, Hong Kong).

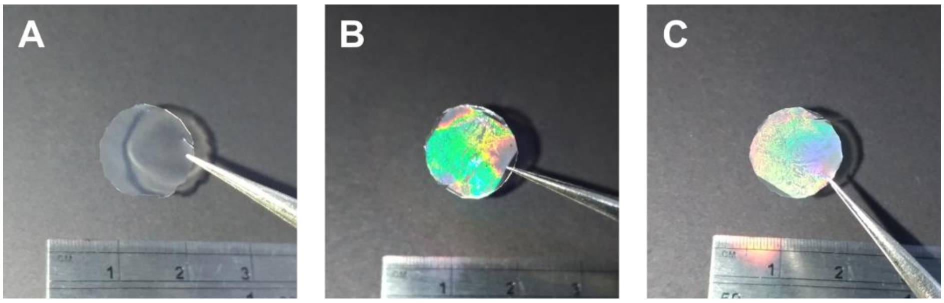

Figure S2: Photographs of the different non-grooved PU substrates: (A) flat, (B) nanopillar, and (C) nanohole. Light was shone on the samples to make the iridescent surface more apparent.

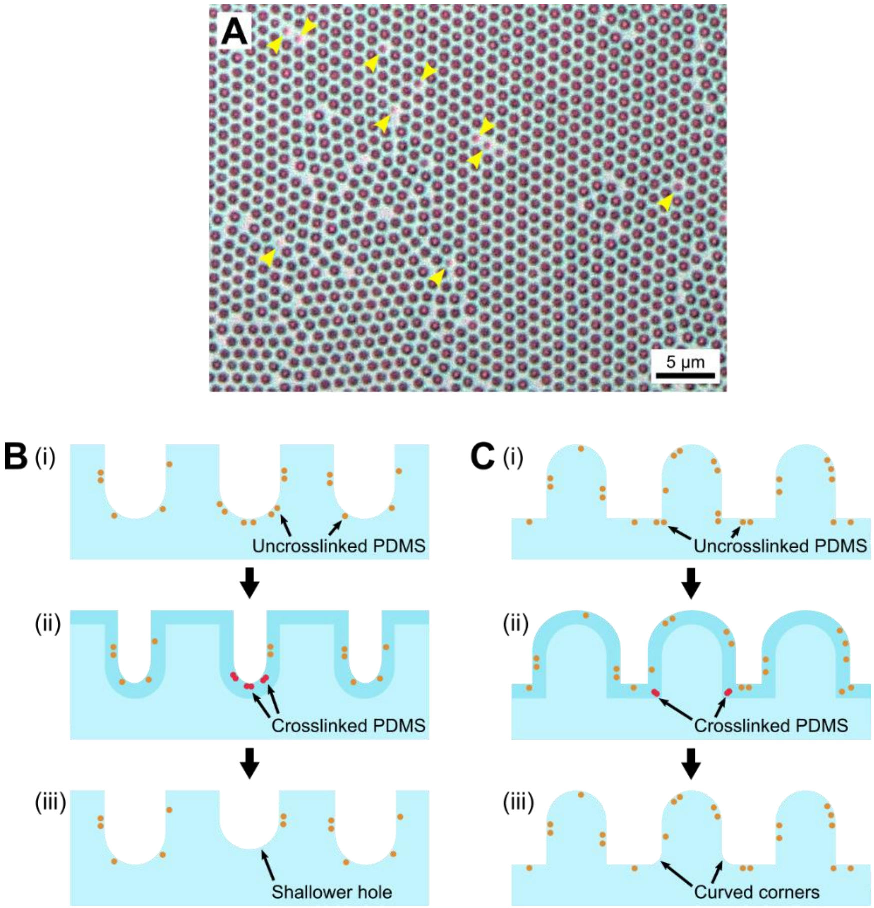

Figure S3: Reduction of PU nanopillar size as viewed under an optical (metallurgical) microscope (indicated by yellow arrowheads; 100× objective; BA310MET; Motic, Hong Kong) (A) and possible occurrences in the molds that cause the phenomenon (B). It is likely that uncrosslinked PDMS monomers were present in the PDMS molds (B, C; i), and during heat treatment in the PU casting process, the PDMS molds expanded and caused closely situated monomers to crosslink (B, C; ii), resulting in changes in the mold topography (B, C; iii). Due to their concave shape, PDMS holes have a higher probability of crosslinking residual monomers (B) than PDMS pillars (C), which results in the observed reduced PU nanopillar sizes.

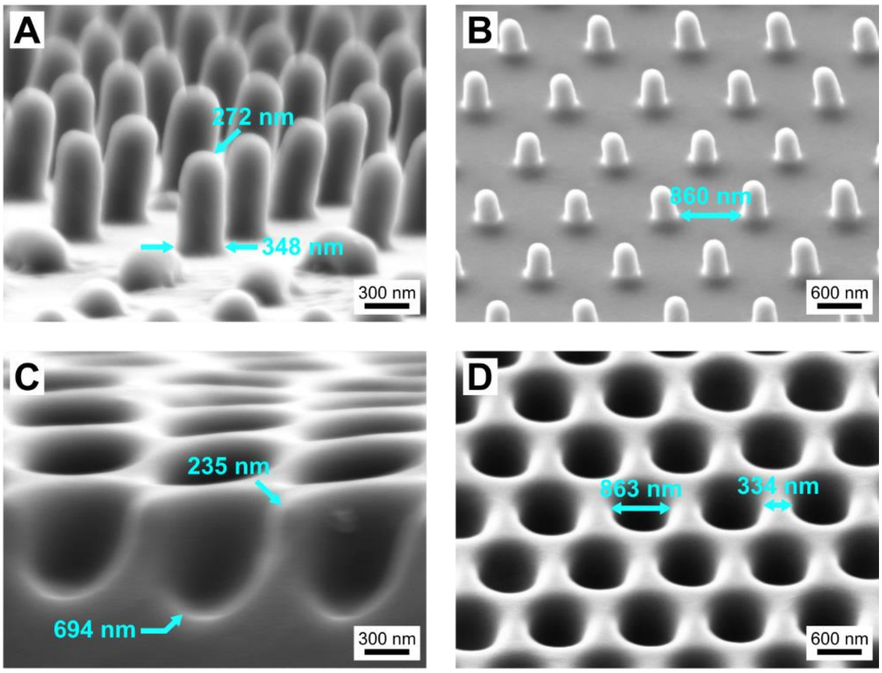

Figure S4: Determination of PU nanostructure dimensions from high-magnification scanning electron micrographs: (A, B) PU nanopillars and (C, D) PU nanoholes. The measurements are also shown in parentheses in Figs. 2E and F of the main article for comparison with atomic force microscopy measurements.

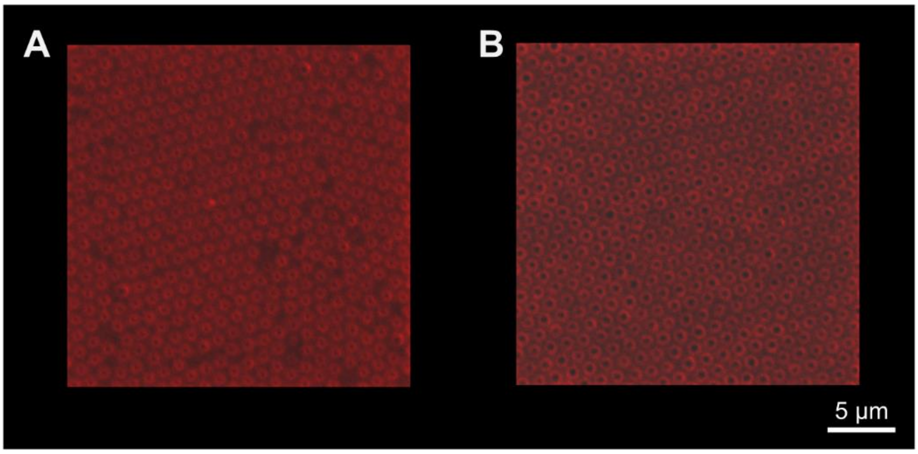

Figure S5: Confocal fluorescence microscopy of immunostained adsorbed laminin on the flat areas of the PU nanopillar (A) and nanohole (B) substrates.

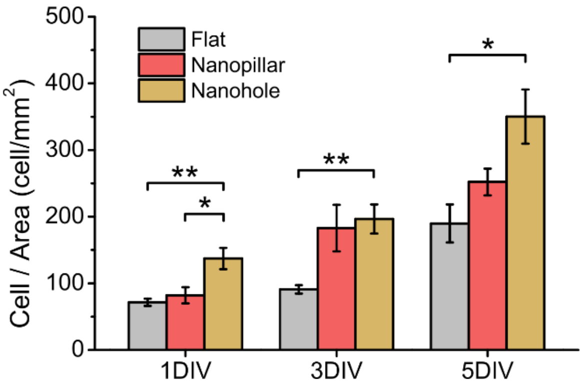

Figure S6: PC12 proliferation on PU flat, nanopillar, and nanohole substrates for 1, 3, and 5 days (*$p < 0.05$; **$p < 0.01$; $n = 9$). Error bars represent the standard error of the mean.

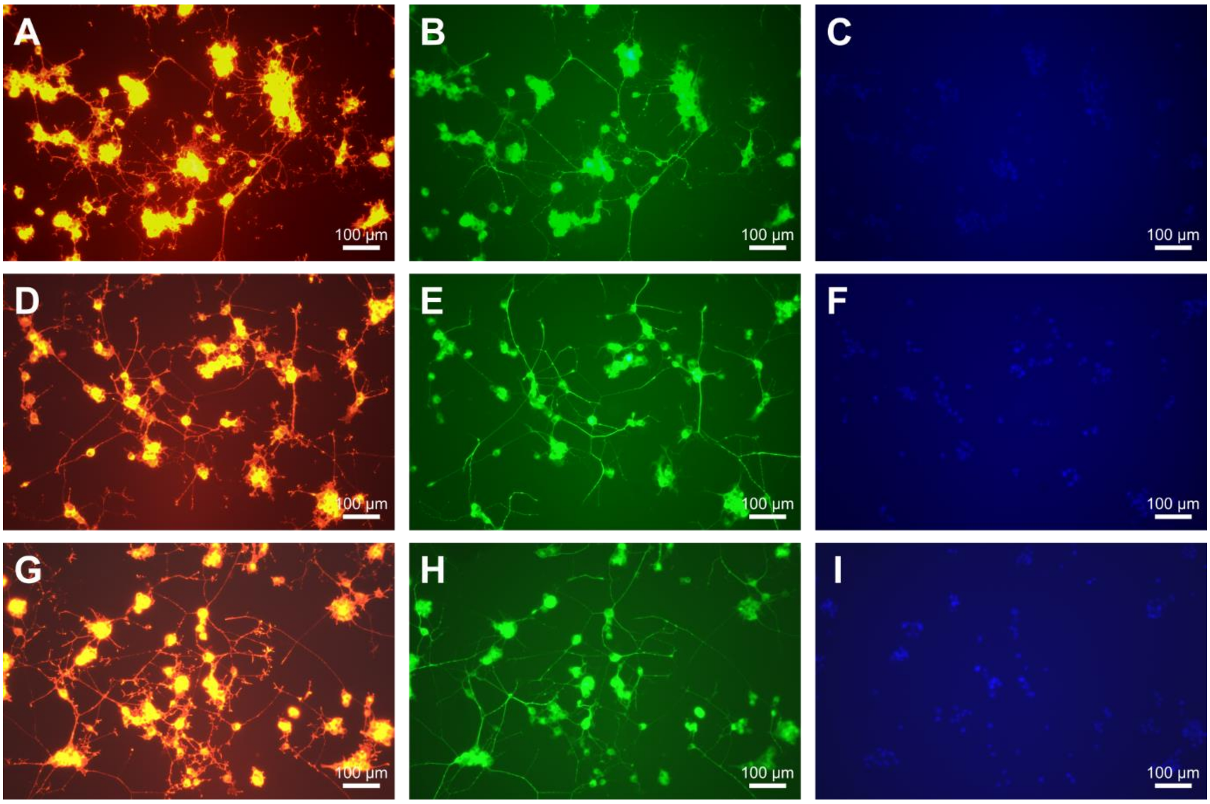

Figure S7: Fluorescence micrographs of PC12 cells on PU flat (A–C), nanopillar (D–F), and nanohole (G–I) substrates, showing the separate channels for visualization of actin (A, D, G), beta-III tubulin (B, E, H), and nucleus (C, F, I) staining.

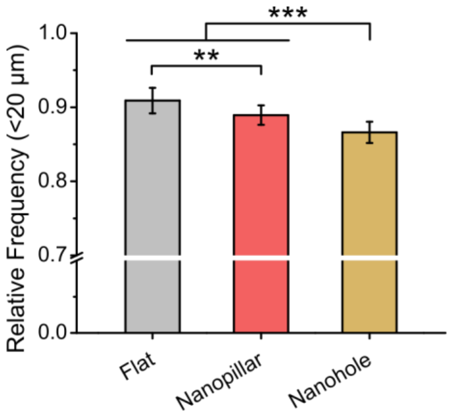

Figure S8: Relative amount of PC12 neurites with branch length less than 20 µm on PU flat, nanopillar, and nanohole substrates (**$p < 0.01$; ***$p < 0.001$; $n = 15$).

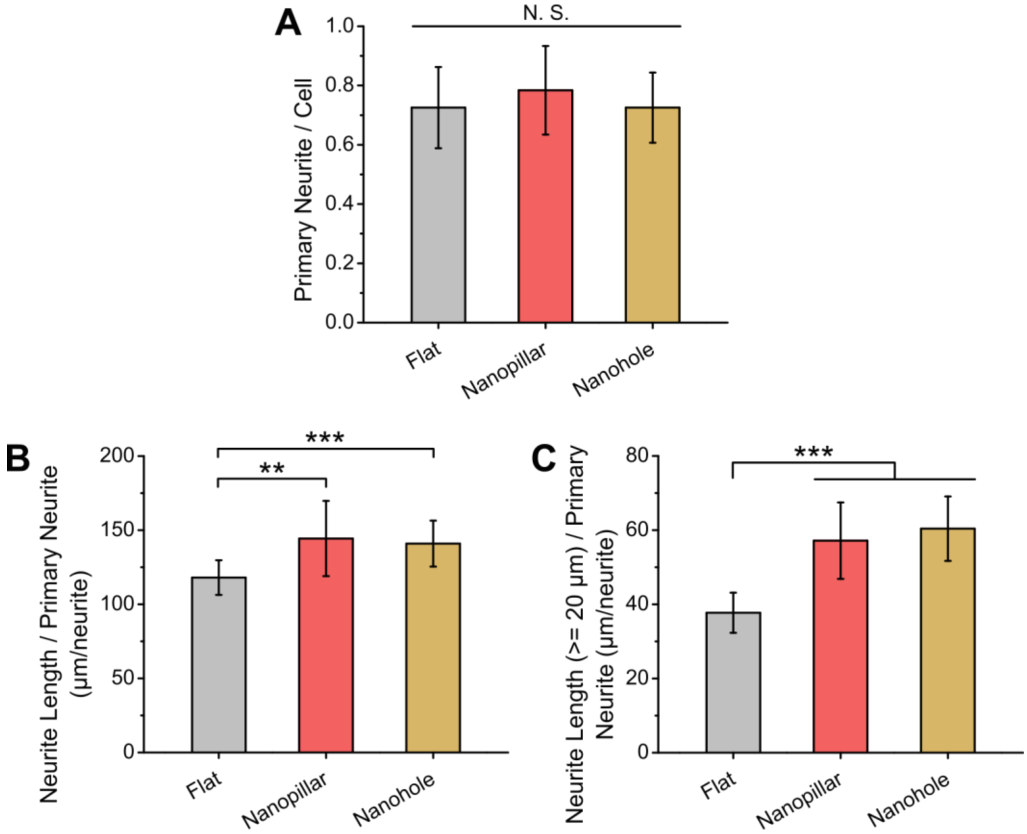

Figure S9: PC12 parameters related to primary neurite count on PU flat, nanopillar, and nanohole substrates: (A) primary neurite count per cell, (B) total neurite length per primary neurite, and (C) total neurite length of branches greater than or equal to 20 µm per primary neurite (N.S. not statistically significant; **$p < 0.01$; ***$p < 0.001$; $n = 15$).

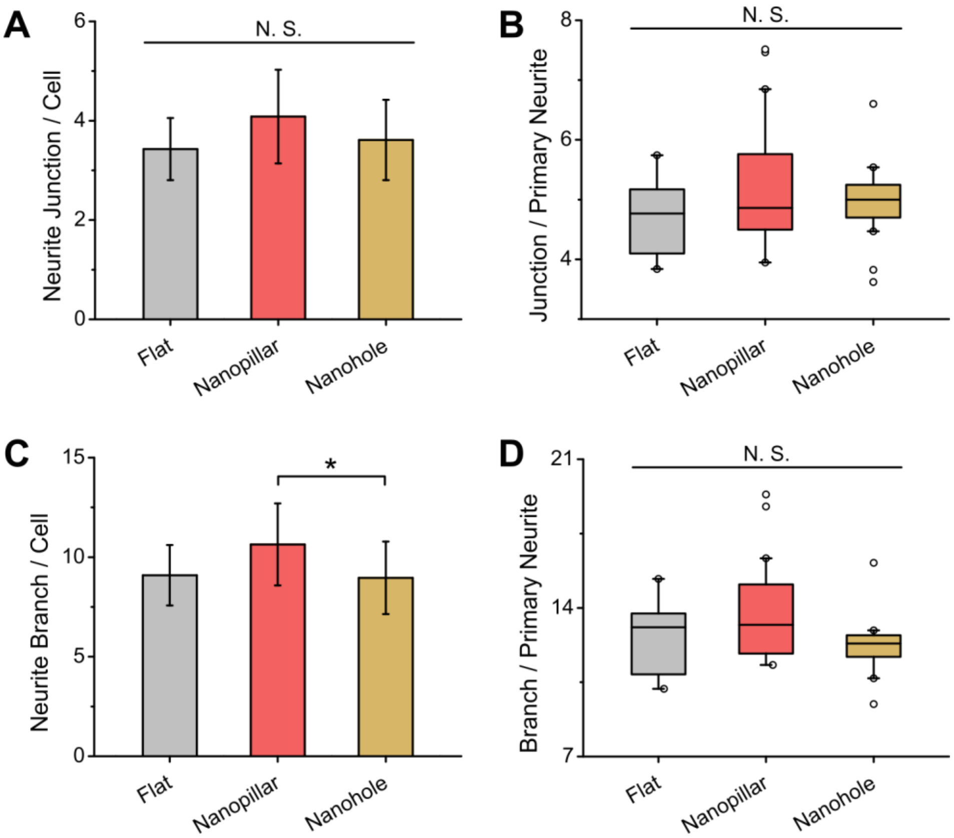

Figure S10: PC12 parameters related to neurite branch and junction counts on PU flat, nanopillar, and nanohole substrates: (A) neurite junction count per cell, (B) neurite junction count per primary neurite, (C) neurite branch count per cell, and (D) neurite branch count per primary neurite (N.S. not statistically significant; *$p < 0.05$; $n = 15$).

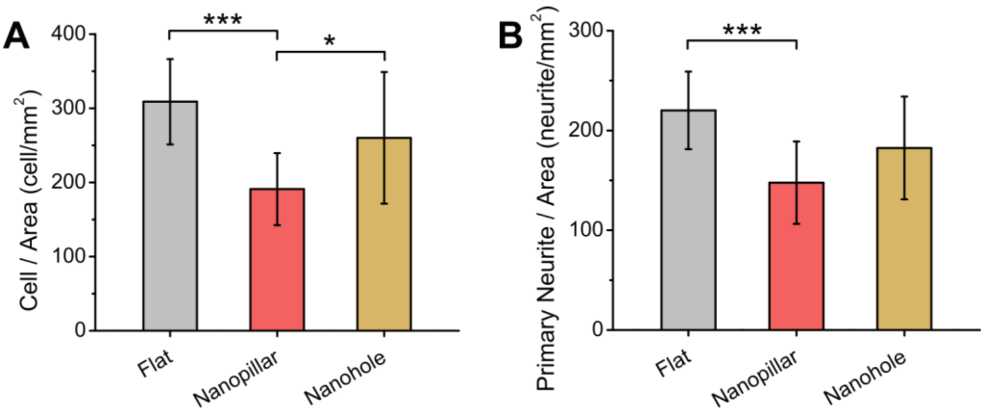

Figure S11: Cell count per unit area (A) and primary neurite count per unit area (B) of PC12 cells on PU flat, nanopillar, and nanohole substrates (*$p < 0.05$; ***$p < 0.001$; $n = 15$).

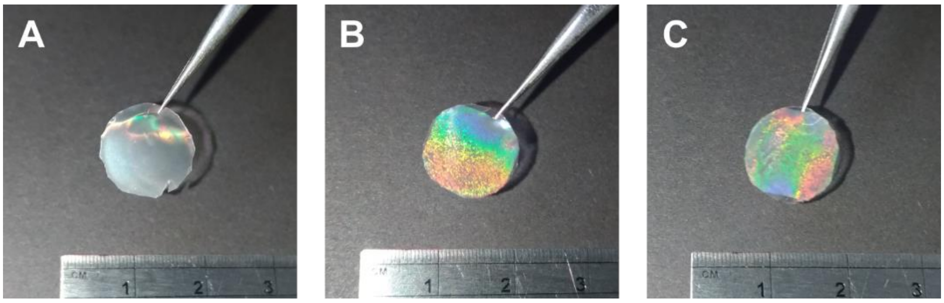

Figure S12: Photographs of the different grooved PU substrates: (A) microgroove, (B) pillar–groove, and (C) hole–groove. Light was shone on the samples to make the iridescent surface more apparent.

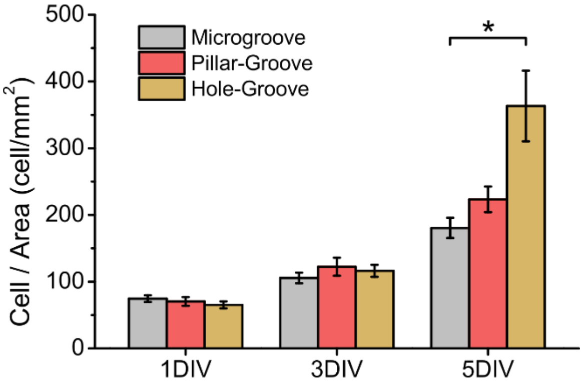

Figure S13: PC12 proliferation on PU microgroove, pillar–groove, and hole–groove substrates for 1, 3, and 5 days (*p < 0.05; n = 9). Error bars represent the standard error of the mean.

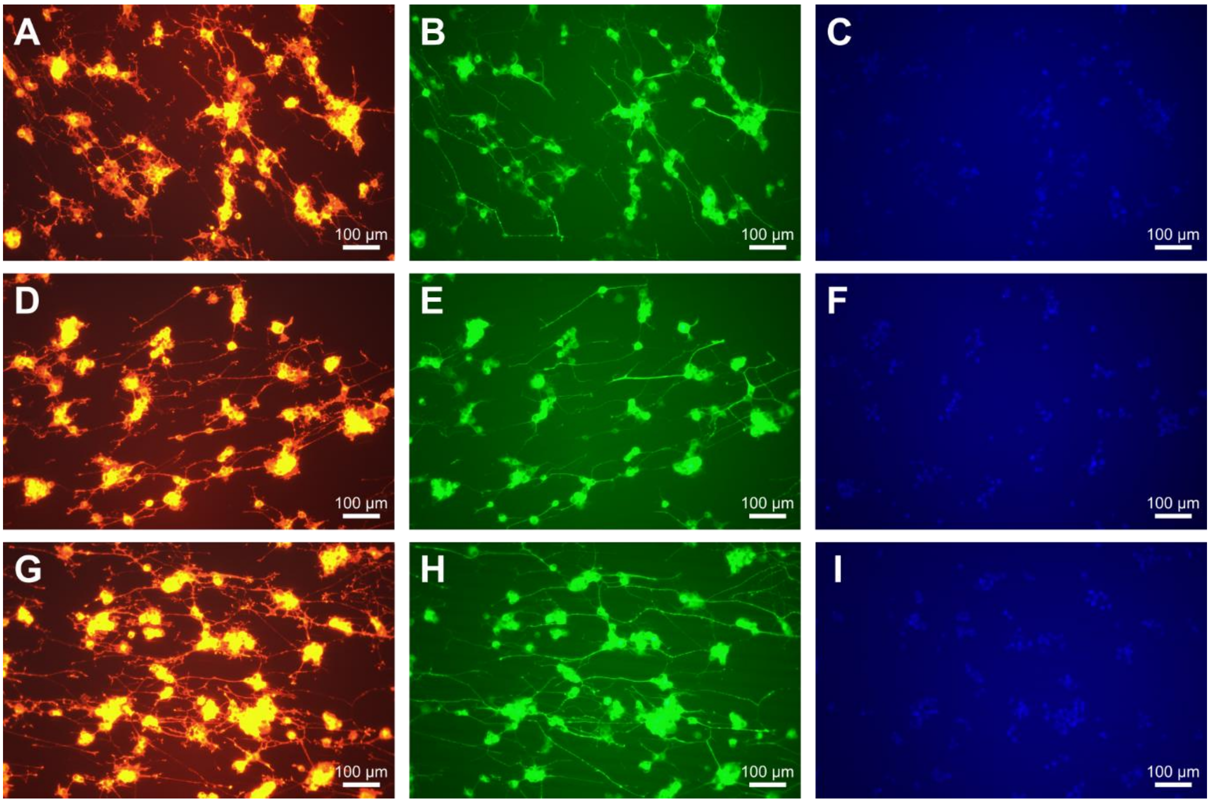

Figure S14: Fluorescence micrographs of PC12 cells on PU microgroove (A–C), pillar–groove (D–F), and hole–groove (G–I) substrates, showing the separate channels for visualization of actin (A, D, G), beta-III tubulin (B, E, H), and nucleus (C, F, I) staining.

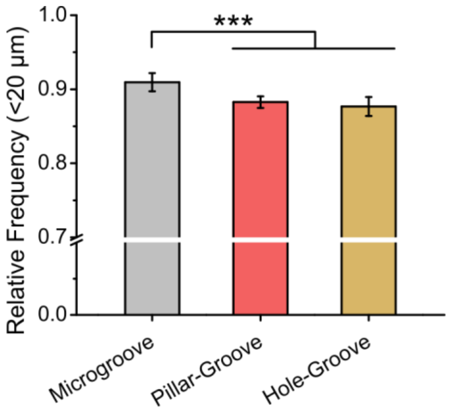

Figure S15: Relative amount of PC12 neurites with branch length less than 20 µm on PU microgroove, pillar–groove, and hole–groove substrates (***p < 0.001; n = 15).

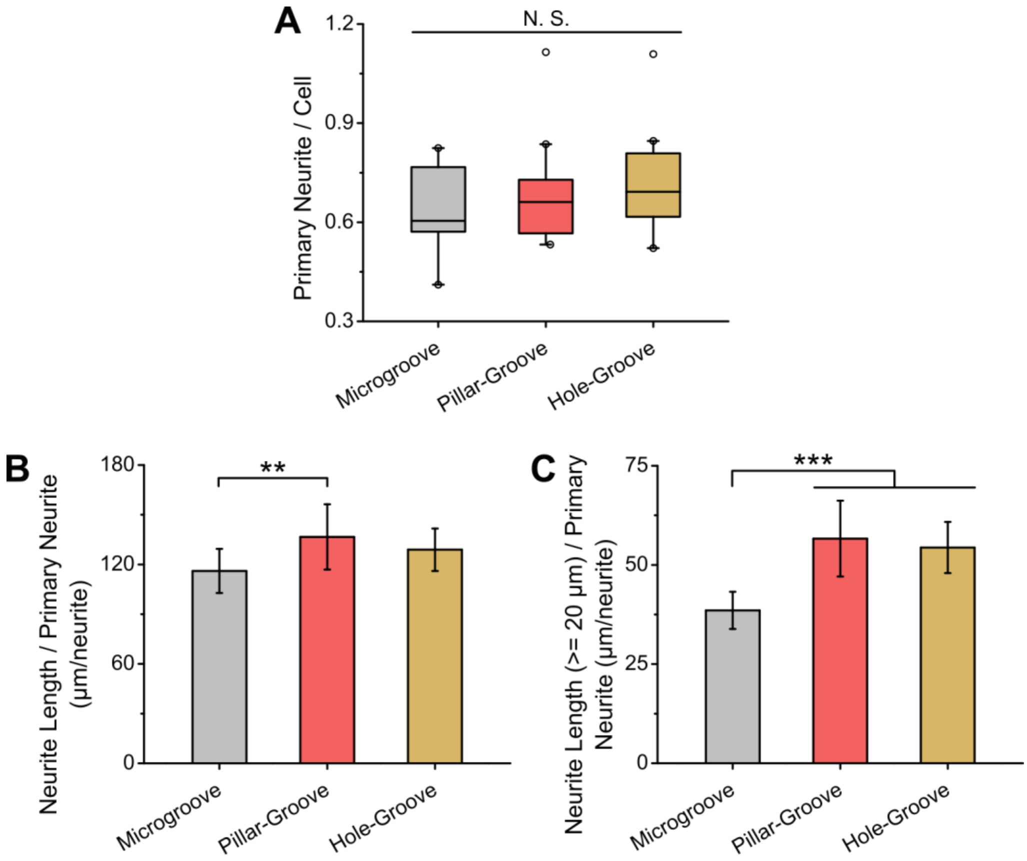

Figure S16: PC12 parameters related to primary neurite count on PU microgroove, pillar–groove, and hole–groove substrates: (A) primary neurite count per cell, (B) total neurite length per primary neurite, and (C) total neurite length of branches greater than or equal to 20 μm per primary neurite (N.S. not statistically significant; $^{**}p < 0.01$; $^{***}p < 0.001$; $n = 15$).

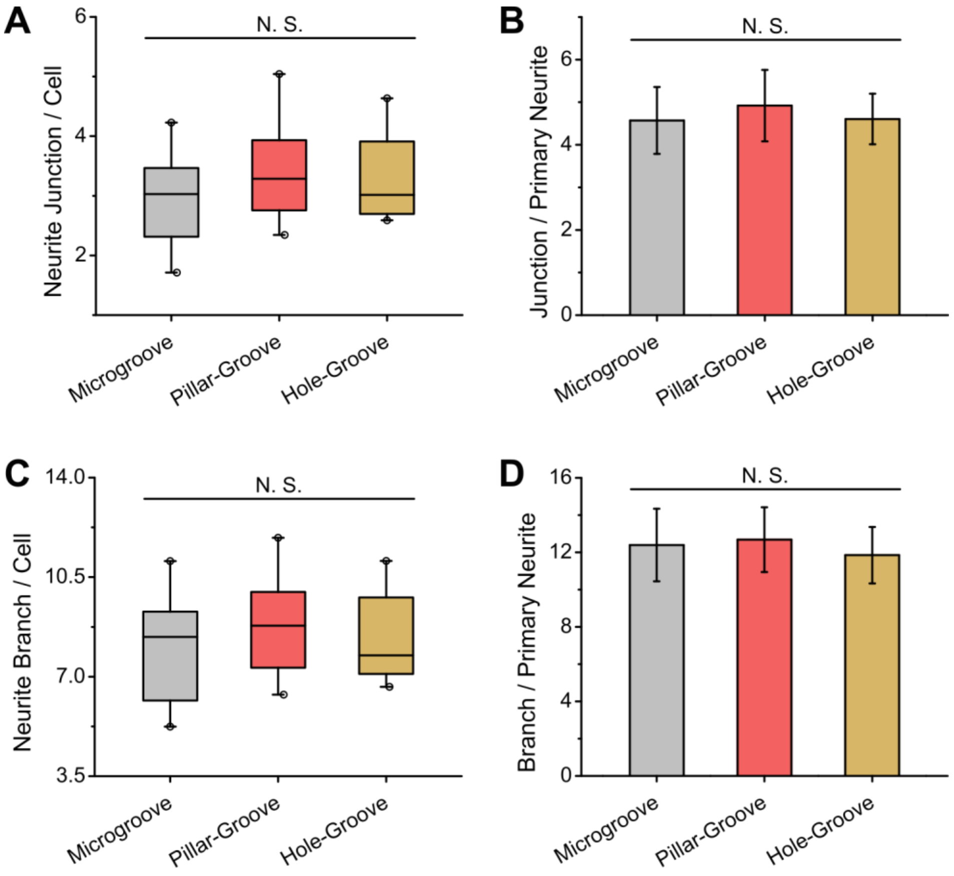

Figure S17: PC12 parameters related to neurite branch and junction counts on PU microgroove, pillar–groove, and hole–groove substrates: (A) neurite junction count per cell, (B) neurite junction count per primary neurite, (C) neurite branch count per cell, and (D) neurite branch count per primary neurite (N.S. not statistically significant; *n* = 15).

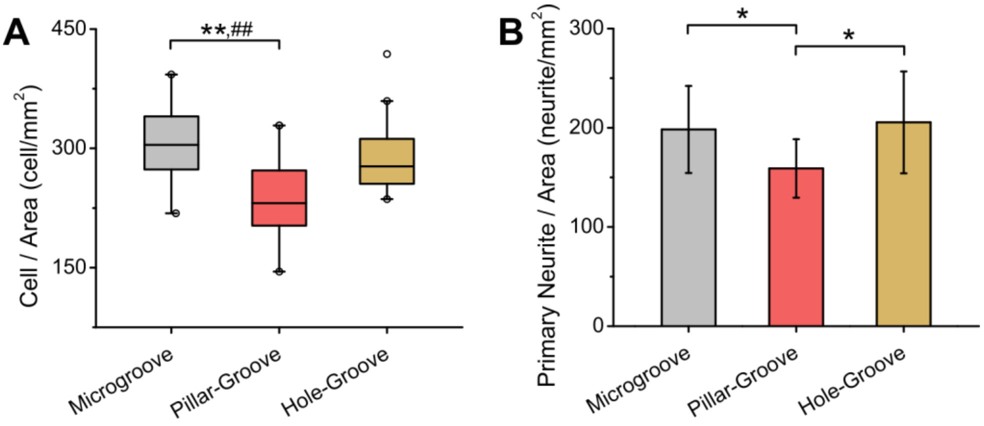

Figure S18: Cell count per unit area (A) and primary neurite count per unit area (B) of PC12 cells on PU microgroove, pillar–groove, and hole–groove substrates (*$p < 0.05$; **,##$p < 0.01$; n = 15). In (A), asterisk (*) and number (#) symbols refer to the distribution and median, respectively.

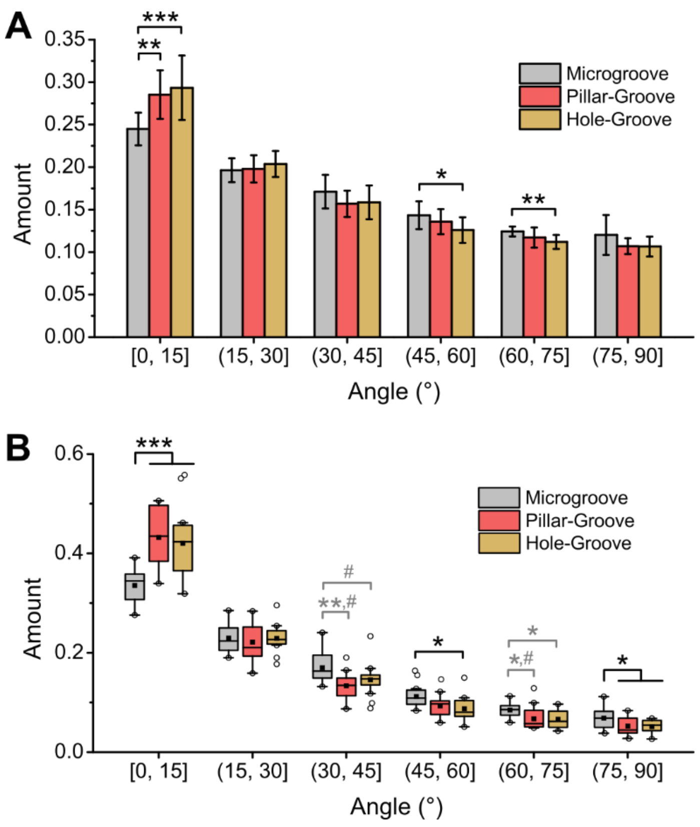

Figure S19: PC12 neurite orientation histogram on PU microgroove, pillar-groove, and hole-groove substrates: (A) all neurite branches and (B) only branches greater than or equal to 20 μm (*,#p < 0.05; **p < 0.01; ***p < 0.001; n = 15). In (B), all the groups have normally distributed data (with the means shown as black-filled squares), except for the hole–groove of (30, 45] and the pillar–groove of (60, 75]. The gray-colored asterisk (*) and number (#) symbols in (B) refer to the distribution and median, respectively.

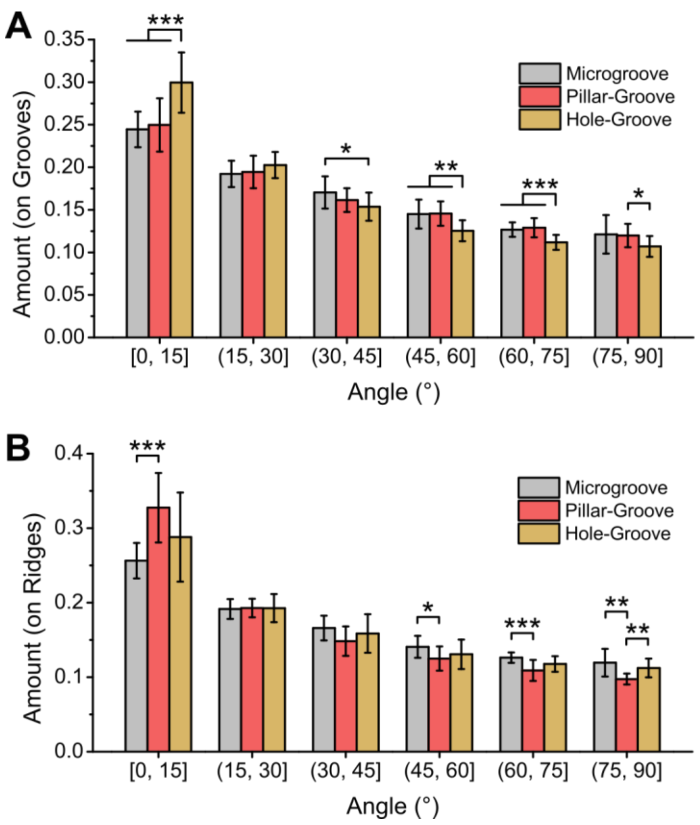

Figure S20: PC12 groove-versus-ridge neurite orientation histogram on PU microgroove, pillar–groove, and hole–groove substrates: (A) microgroove areas and (B) microridge areas (*p < 0.05; **p < 0.01; ***p < 0.001; n = 15).

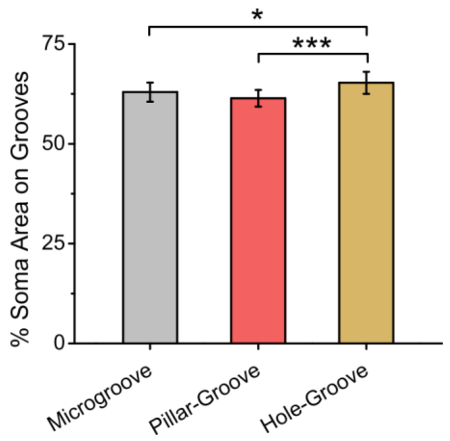

Figure S21: Percentage of PC12 soma area on microgroove areas on PU microgroove, pillar–groove, and hole–groove substrates (*p < 0.05; ***p < 0.001; n = 15).

References

1. Schindelin, J.; Arganda-Carreras, I.; Frise, E.; Kaynig, V.; Longair, M.; Pietzsch, T.; Preibisch, S.; Rueden, C.; Saalfeld, S.; Schmid, B.; Tinevez, J.-Y.; White, D. J.; Hartenstein, V.; Eliceiri, K.; Tomancak, P.; Cardona, A. *Nat. Methods* **2012**, *9* (7), 676-682.
2. Vinzons, L. U.; Lin, S.-P. *ACS Appl. Nano Mater.* **2022**, *5* (5), 6935-6953.
3. Pool, M.; Thiemann, J.; Bar-Or, A.; Fournier, A. E. *J. Neurosci. Methods* **2008**, *168* (1), 134-139.
4. Ho, S.-Y.; Chao, C.-Y.; Huang, H.-L.; Chiu, T.-W.; Charoenkwan, P.; Hwang, E. *BMC Bioinf.* **2011**, *12* (1), 230.
